# Elucidating the cellular determinants of targeted membrane protein degradation by lysosome-targeting chimeras

**DOI:** 10.1126/science.adf6249

**Published:** 2023-10-20

**Authors:** Green Ahn, Nicholas M. Riley, Roarke A. Kamber, Simon Wisnovsky, Salvador Moncayo von Hase, Michael C. Bassik, Steven M. Banik, Carolyn R. Bertozzi

**Affiliations:** 1 Department of Chemistry, Stanford University, Stanford, CA 94305, USA.; 2 Sarafan ChEM-H, Stanford University, Stanford, CA 94305, USA.; 3 Department of Genetics, Stanford University, Stanford, CA 94305, USA.; 4 Pharmaceutical Sciences, University of British Columbia, Vancouver, BC, Canada.; 5 Howard Hughes Medical Institute, Stanford University, Stanford, CA 94305, USA.

## Abstract

Targeted protein degradation can provide advantages over inhibition approaches in the development of therapeutic strategies. Lysosome-targeting chimeras (LYTACs) harness receptors, such as the cation-independent mannose 6–phosphate receptor (CI-M6PR), to direct extracellular proteins to lysosomes. In this work, we used a genome-wide CRISPR knockout approach to identify modulators of LYTAC-mediated membrane protein degradation in human cells. We found that disrupting retromer genes improved target degradation by reducing LYTAC recycling to the plasma membrane. Neddylated cullin-3 facilitated LYTAC-complex lysosomal maturation and was a predictive marker for LYTAC efficacy. A substantial fraction of cell surface CI-M6PR remains occupied by endogenous M6P-modified glycoproteins. Thus, inhibition of M6P biosynthesis increased the internalization of LYTAC-target complexes. Our findings inform design strategies for next-generation LYTACs and elucidate aspects of cell surface receptor occupancy and trafficking.

## Abstract (summary)

**INTRODUCTION:**

Repurposing endogenous cellular machinery to degrade disease-driving proteins is a promising therapeutic strategy, as evidenced by the rapid development of intracellular degraders over the past two decades. Extracellular degraders, including lysosome-targeting chimeras (LYTACs), have expanded the scope of protein degradation to secreted and plasma membrane targets. LYTACs harness endogenous cell surface receptors, such as cation-independent mannose 6–phosphate receptor (CI-M6PR), to traffic specific proteins between the plasma membrane and lysosomes. However, the cellular features that affect the ability to hijack lysosomal trafficking for membrane protein degradation are largely unknown. An approach to map features that potentiate or attenuate the ability to degrade membrane proteins is needed to further understand extracellular degradation mechanisms.

**RATIONALE:**

The factors that affect membrane protein degradation could facilitate rational therapeutic development and reveal biological insights crucial for numerous therapeutic modalities. Membrane proteins, unlike soluble secreted proteins, can retain their own distinct trafficking behavior through the endosomal system, which must be overcome or enhanced for targeted degradation by extracellular degraders. We deploy an unbiased genome-wide CRISPR knockout screening approach complemented by proteomics to identify and characterize cellular components that modulate LYTAC-mediated membrane protein degradation. This approach combines modular chemically defined LYTAC ligands, scalable cell separation through magnetic beads, immunoprecipitation, and cell surface proteomics to identify key molecular players.

**RESULTS:**

This genome-wide screen confirmed the importance of several endolysosomal genes for LYTAC-mediated degradation. We characterized the effect of additional genes distinct from the canonical endolysosomal pathway or genes whose knockout can enhance LYTAC activity. We identified that the retromer complex recycles LYTAC–CI-M6PR complexes from endosomes to the cell surface before dissociation of LYTAC from the receptor. Disruption of retromer complex genes enhanced degradation mediated by LYTAC by inhibiting this competitive recycling pathway. Genes involved in the neddylation of cullin 3 (CUL3), and therefore activation of its E3 ligase activity, were crucial for the trafficking of LYTAC-target complexes to lysosomes. We determined that levels of neddylated CUL3 are correlated with degradation efficacy, thereby serving as a potential predictive marker for LYTAC activity. In addition, we revealed that genes involved in the mannose 6–phosphate (M6P) biosynthesis pathway counteract LYTAC activity through synthesizing competitive ligands for CI-M6PR. We observed that cell surface CI-M6PR receptors are occupied by distinct M6P-modified lysosomal glycoproteins. Disruption of M6P biosynthesis increased the fraction of unoccupied receptors on the cell surface, allowing enhanced LYTAC-receptor engagement and internalization.

**CONCLUSION:**

Mechanistic studies driven by extracellular degraders can reveal previously unknown aspects of receptor and endosomal biology. This study uncovered drivers of extracellular degradation using CI-M6PR as a lysosome trafficking receptor. The findings of this study motivate new design strategies for lysosome-targeted protein degraders. Furthermore, the magnetic enrichment–based genomic screen approach can be expanded to any other extracellular degraders, allowing the identification of common and distinct mechanisms across extracellular degradation platforms. We anticipate that the impacts of receptor recycling, occupancy, and ability to traverse the endolysosomal system revealed in our work will have broader implications for other therapeutics that rely on cell surface–to–lysosome trafficking, such as enzyme-replacement therapies, nucleic acid delivery, and antibody-drug conjugates.

## Editor’s summary

Delivery to lysosomes of various biotherapeutics such as lysosomal enzyme replacement therapies, antibody-drug conjugates, and oligonucleotide therapeutics is crucial for their activity. Extracellular protein degraders, including lysosome-targeting chimeras (LYTACs), enable the precise depletion of proteins from the extracellular space through lysosomal targeting. Ahn *et al*. used unbiased genetic screens and proteomics to map cellular determinants of extracellular degrader activity. Activation of the E3 Cullin-3 enabled LYTAC target complexes to reach lysosomes and was a predictive marker for cell susceptibility to degraders. Cell surface CI-M6PR, a prototypical lysosome-targeting receptor, was found to be blocked by endogenous mannose-6-phosphate (M6P)–modified glycoproteins. The retromer complex counteracted this degradation activity through recycling LYTAC-CI-M6PR complexes. Accordingly, disruption of M6P biosynthesis or CI-M6PR recycling increased LYTAC activity. Detailed mechanistic study of membrane protein degradation by LYTACs can thus elucidate endocytic biology and could help in the rational deployment of degraders against disease. —Stella M. Hurtley

## Graphical Abstract

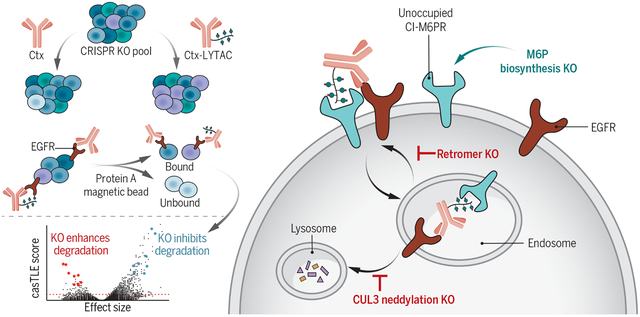

**Identifying cellular determinants of LYTAC-mediated degradation of membrane proteins.** A genome-wide CRISPR screen was conducted to identify factors regulating LYTAC-mediated degradation of epidermal growth factor receptor (EGFR) via magnetic cell sorting. Knockout (KO) of retromer complex resulted in reduced recycling of LYTAC-target complex. CUL3 neddylation was essential for lysosomal trafficking and degradation. Disruption of M6P biosynthesis pathway resulted in increased unoccupied CI-M6PR on the cell surface, enhancing LYTAC-receptor internalization. Ctx, cetuximab.

Selective depletion of individual proteins is a validated approach for modulating numerous disease-driving targets. Intracellular degradation approaches using proteolysis-targeting chimeras (PROTACs) (1, 2) or molecular glues (3, 4), both of which recruit E3 ligases to promote ubiquitination of target proteins, have already demonstrated clinical impact (5, 6). Technologies for targeted degradation of extracellular proteins, including lysosome-targeting chimeras (LYTACs) (7, 8), molecular degraders of extracellular proteins through the asialoglycoprotein receptor (MoDE-As) (9), antibody-based PROTACs (AbTACs) (10), proteolysis-targeting antibodies (PROTABs) (11), and cytokine receptor–targeting chimeras (KineTACs) (12), have recently expanded the scope of potential therapeutic targets. A common theme of these approaches is harnessing the cellular internalization machinery to drive extracellular protein degradation in the lysosome. For example, LYTACs are bifunctional molecules that form ternary complexes with a protein of interest and a lysosome trafficking receptor, such as the cation-independent mannose 6–phosphate receptor (CI-M6PR) (7) (Fig. 1A) or the liver-restricted asialoglycoprotein receptor (ASGPR) (8, 13).

The development of PROTACs and molecular glues has been accelerated by mechanistic studies that have revealed key cellular determinants for induced protein degradation (14–16). However, the cellular features that enable or inhibit membrane protein degradation remain unclear. An understanding of the mechanisms driving targeted degradation of extracellular and transmembrane proteins could potentially accelerate therapeutic development and application. Given the importance of lysosomal trafficking for the delivery of various biotherapeutics, including lysosomal enzyme replacement therapies (ERTs) (17, 18), antibody-drug conjugates (ADCs) (19, 20), and oligonucleotide therapeutics (21–23), characterizing the mechanisms of extracellular degraders is likely to reveal principles that can benefit numerous therapeutic modalities. In this work, we performed a genome-wide CRISPR knockout (KO) screen to identify essential determinants of CI-M6PR–LYTAC–mediated lysosomal trafficking and degradation of membrane proteins.

## Design of next-generation LYTACs comprising antibody-glycopeptide conjugates

**Fig. 1.**
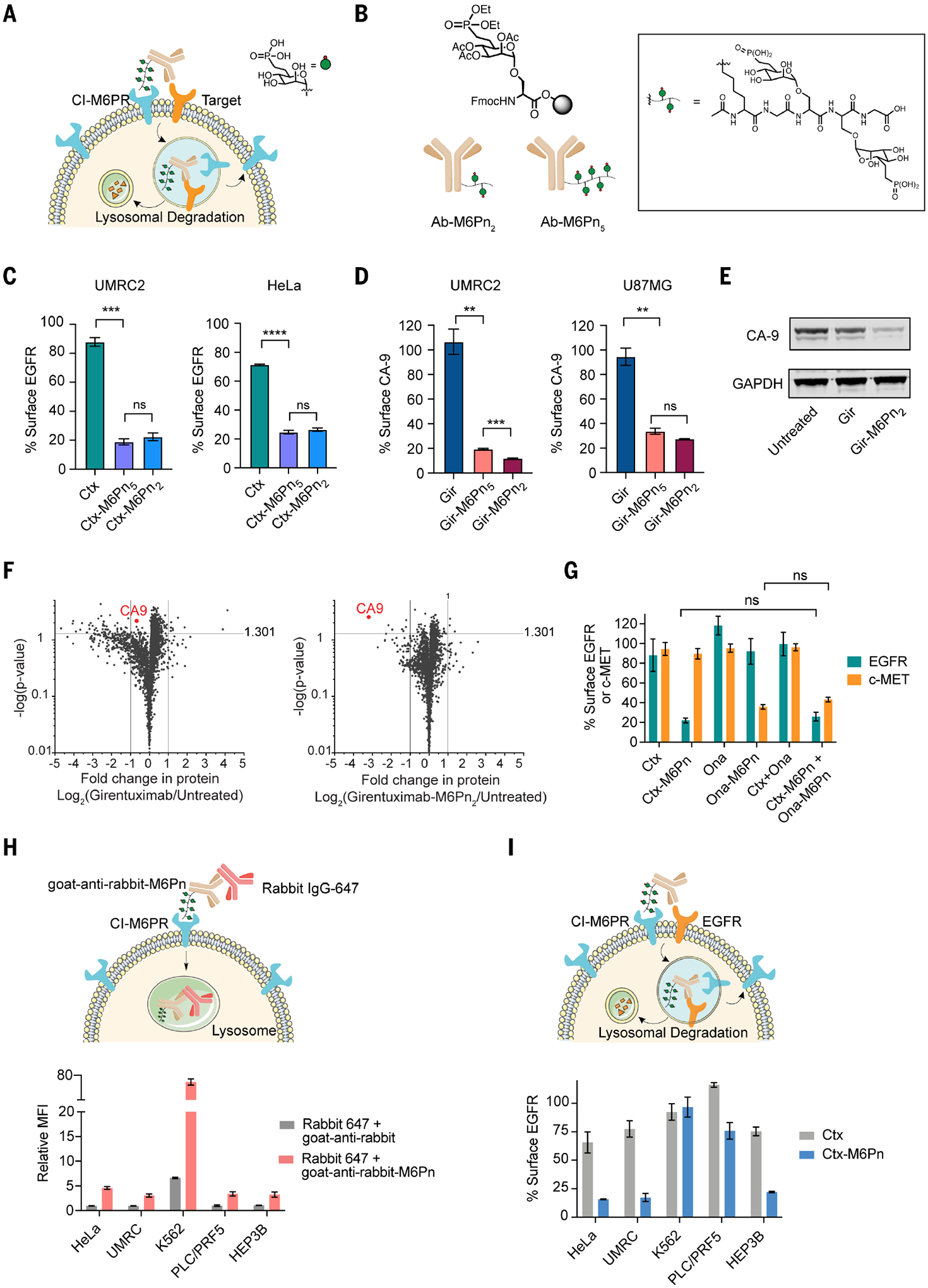
LYTACs comprising antibody-glycopeptide conjugates enable degradation of membrane targets. (**A**) Membrane protein degradation mediated by LYTACs that harness CI-M6PR. (**B**) Synthesis of homogeneous M6Pn ligands through solid-phase peptide synthesis (SPPS). (**C**) Degradation of EGFR with M6Pn-LYTACs in UMRC2 and HeLa cells as determined by live-cell flow cytometry after 48 hours of treatment with 10 nM Ctx or LYTACs. (**D**) Degradation of CA9 in UMRC2 and U87MG cells as determined by live-cell flow cytometry after 48 hours of treatment with 10 nM Gir or LYTACs. (**E**) Immunoblot analysis of CA9 levels in UMRC2 cells after treatment with 10 nM Gir or Gir- M6Pn_2_ for 48 hours. GAPDH, glyceraldehyde-3-phosphate dehydrogenase. (**F**) Fold change in the abundance of proteins in UMRC2 cells detected by quantitative proteomics analysis after 48 hours of treatment with 10 nM Gir or Gir-M6Pn_2_. (**G**) Degradation of cell surface c-MET and EGFR in UMRC2 cells as determined by live-cell flow cytometry after 48 hours of single treatment with 10 nM Ctx or Ona-M6Pn or cotreatment of Ctx and Ona-M6Pn. (**H**) LYTAC-mediated uptake of rabbit IgG-647 in various cell lines. Mean fluorescence intensity (MFI) relative to the control (rabbit IgG-647 only) for cells incubated at 37°C for 1 hour with 50 nM rabbit IgG-647 and 25 nM goat anti-rabbit or goat anti-rabbit M6Pn. MFI was determined by live-cell flow cytometry. (**I**) Degradation of cell surface EGFR in various cell lines as determined by live-cell flow cytometry after 48 hours of treatment with 10 nM Ctx or Ctx conjugates. For (C), (D), and (F) to (I), data represent three independent experiments, and data are shown as means ± SEMs. *P* values were determined by unpaired two-tailed *t* tests. NS, not significant; *P ≤ 0.05; **P ≤ 0.01; ***P ≤ 0.001; ****P ≤ 0.0001.

To initiate a mechanistic study, we first developed synthetically scalable next-generation LYTAC molecules. Synthesis of first-generation CI-M6PR LYTACs used NCA (*N*-carboxyanhy-dride) polymerization to generate mixtures of alanine–serine–mannose 6–phosphonate (M6Pn) copolymers with various lengths and compositions (7). The heterogeneity of the initial M6Pn-glycopolymer could result in batch-to-batch differences and prevented characterization of the precise molecular nature of the LYTAC molecule. We developed homogeneous peptides to maintain control over structural composition of the antibody-peptide conjugates. We established a synthetic route to synthesize Fmoc-Serine(M6Pn)-OH monomer that could be easily incorporated into a standard solid-phase peptide synthesis (SPPS) workflow with high yield (fig. S1). Glycopeptides bearing two M6Pn (M6Pn_2_) and five M6Pn (M6Pn_5_) residues were readily conjugated to cetuximab (Ctx), the clinically approved anti–epidermal growth factor receptor (EGFR) antibody, using Copper-free click chemistry (Fig. 1B and fig. S2). Ctx-M6Pn_5_ and Ctx-M6Pn_2_ degraded ~80% of cell surface EGFR in UMRC2 and HeLa cells (Fig. 1C and fig. S3A). Maximum degradation was achieved at 10 nM for both Ctx-M6Pn_5_ and Ctx-M6Pn_2_, and higher concentrations did not reduce degradation (fig. S3C). More than 50% of the total EGFR was degraded after 24 hours of treatment in UMRC2 and HeLa cells (fig. S3, D and E). The presence or absence of serum did not affect the degradation efficacy (fig. S3F). We used a fluorophore-labeled rabbit immunoglobulin G (IgG) as a cargo in the presence of goat-anti-rabbit (control) or goat-anti-rabbit-M6Pn_5_ (LYTAC) to evaluate internalization of a soluble protein. The half maximal inhibitory concentration (IC_50_) for monomeric M6P inhibition of IgG uptake by goat-anti-rabbit-M6Pn_5_ was 4.3 mM for LYTAC-mediated internalization (fig. S3G), and saturation of cargo uptake was reached at 100 nM LYTAC (fig. S3H).

To expand the targetable membrane protein scope, we conjugated the peptides to girentuximab (Gir) and onartuzumab (Ona)—antibodies against carbonic anhydrase IX (CA9) and c-Met, respectively. Gir-M6Pn_5_ and Gir-M6Pn_2_ degraded >80% of surface CA9 in UMRC2 cells and 70 to 80% in U87MG cells (Fig. 1D and fig. S3B). Only minor differences in efficacy were observed between M6Pn_2_ and M6Pn_5_, although Gir-M6Pn_2_ conjugates were optimal for CA9 degradation, and Ctx-M6Pn_5_ conjugates were optimal for EGFR degradation. We confirmed that Gir-M6Pn_2_ degraded CA9 (Fig. 1E) and demonstrated that the down-regulation was highly selective for CA9 by quantitative proteomics (Fig. 1F). Given that only a small percentage of CI-M6PR is recycled to the plasma membrane, we investigated whether there is a capacity limit to CI-M6PR–induced degradation by assessing whether we could degrade two different membrane targets simultaneously. Ona-M6Pn_5_ drove degradation of c-MET, and cotreatment with Ona-M6Pn_5_ and Ctx-M6Pn_5_ resulted in coincident degradation of EGFR and c-MET in two cell lines (Fig. 1G and fig. S4). We did not observe a loss of degradation efficacy with dual treatments, which suggests that the capacity for CI-M6PR–mediated internalization is not saturated through LYTAC appropriation.

We evaluated soluble cargo internalization using fluorophore-labeled rabbit IgG with a M6Pn_5_-conjugated goat-anti-rabbit (LYTAC) across five cell lines. K562 had significantly higher LYTAC-mediated internalization, whereas the other cell lines demonstrated low to moderate uptake capacity (Fig. 1H). The trend for internalization of soluble cargo correlated with cell surface CI-M6PR expression levels across the cell lines (fig. S5). We asked whether successful uptake of soluble cargo predicted successful degradation of membrane proteins. Although HeLa, UMRC2, and HEP3B showed efficient surface degradation of EGFR, they exhibited poor soluble cargo internalization capacity. No EGFR degradation was observed upon LYTAC treatment in K562 cells (Fig. 1I). The lack of correlation between the uptake of soluble cargo and membrane protein degradation suggests that regulators of induced membrane protein degradation are different from those for soluble cargo uptake. A previous CRISPRi screen had determined essential genes for uptake of soluble cargo (NeutrAvidin-647) by biotinylated poly(M6Pn) (7). Given the disconnect between soluble protein uptake and membrane protein degradation efficiency, we reasoned that features that drive the two processes might differ. Membrane proteins play critical roles in tumorigenesis and neurological diseases (24, 25), and the ability to degrade them in various contexts would be a beneficial therapeutic approach. A mechanistic understanding of induced membrane protein degradation could expand and generalize the technology while providing insight into endocytic regulators.

## A magnetic cell sorting–based genome-wide CRISPR screen identifies key regulators of LYTAC-mediated degradation

**Fig. 2.**
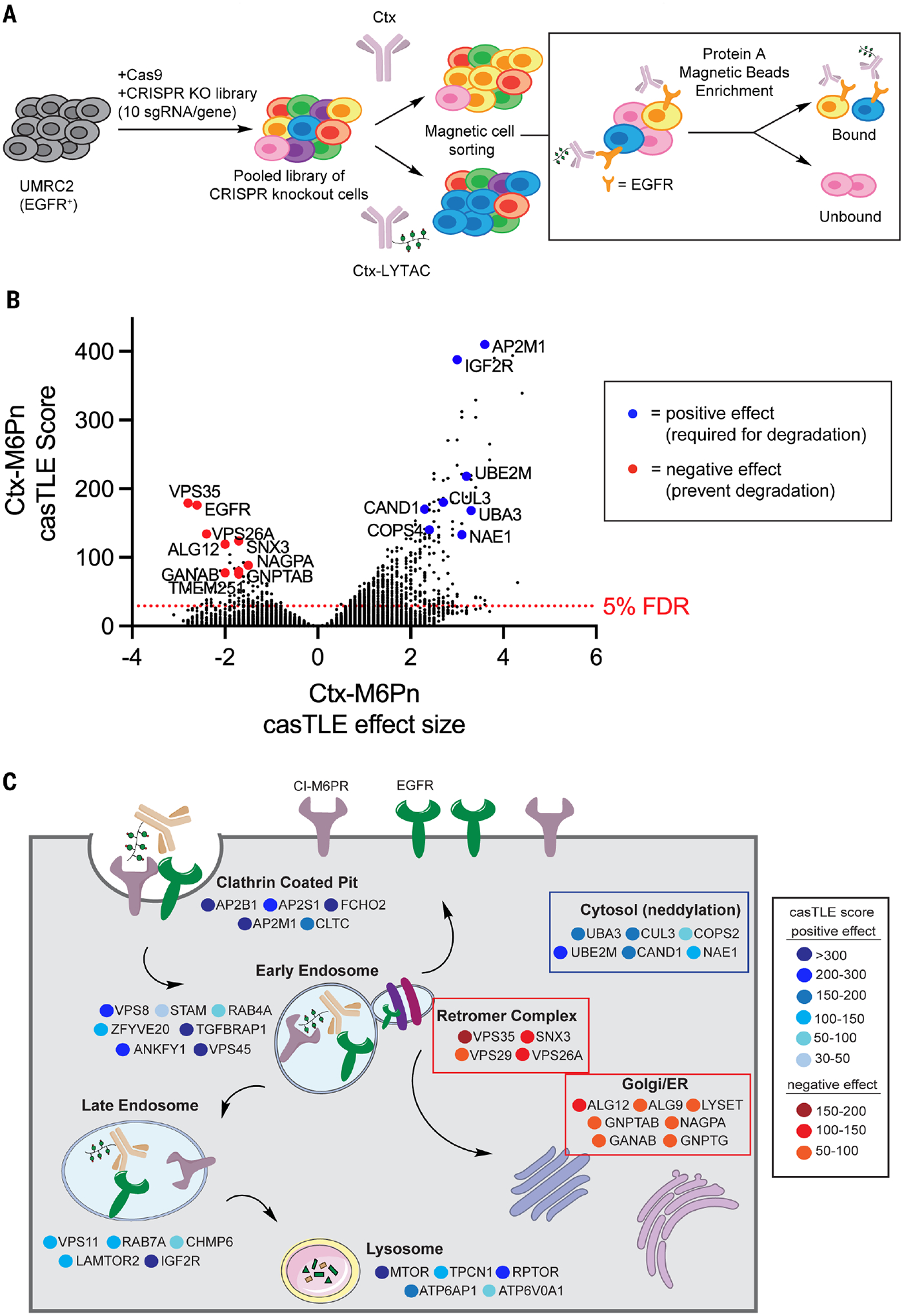
Genome-wide CRISPR KO screen identifies regulators for LYTAC-mediated membrane protein degradation. (**A**) MACS-based CRISPR KO screen in UMRC2 cells stably expressing Cas9 and a library of sgRNAs with a genome-wide coverage. Cells were treated with Ctx or Ctx-LYTAC for 48 hours and enriched for bound and unbound fractions using protein A magnetic beads. (**B**) Selected gene hits for regulation of EGFR degradation by Ctx-M6Pn. Hits with positive effect are in blue, and hits with negative effect are in red. (**C**) Schematic of hits categorized by subcellular localization or processes according to GO annotations, color coded by casTLE score. Genes with positive effect are in blue, and genes with negative effect are in red.

Magnetic cell sorting (MACS) approaches enable rapid sorting of adherent cells while maintaining high cell viability (26–28). To provide insight into membrane protein degradation, we designed a genome-wide CRISPR deletion screen using magnetic enrichment. We generated UMRC2-Cas9 cells infected with a genome-wide single-guide RNA (sgRNA) knockout library (29) and treated cells with Ctx or Ctx-M6Pn. Populations that lost EGFR expression on the cell surface were isolated by sequestering EGFR-positive cells with protein A magnetic beads, which bind to the Fc region of human antibodies (Ctx). No differences in protein A binding between Ctx and Ctx-M6Pn were observed. Cells treated with Ctx-M6Pn that lost the ability to degrade EGFR through CRISPR-mediated gene knockout were retained in the bound fraction, whereas cells that lost cell surface EGFR remained in the unbound fraction. These enriched fractions were analyzed by next-generation sequencing to identify the genes required for LYTAC-mediated membrane protein degradation (Fig. 2A). Analysis and comparison of the guide compositions were performed using casTLE (30). Genes with positive effect size potentiate degradation and have higher sgRNA enrichment in the bound fraction. Genes with negative effect size attenuate degradation and have less abundant sgRNAs in the bound fraction (Fig. 2B). *IGF2R*, an alternative gene name for CI-M6PR, was a top hit with a positive effect size from the Ctx-M6Pn screen, as expected because M6Pn-based LYTACs rely on the binding and trafficking of CI-M6PR. Ctx alone marginally down-regulated EGFR, but the genes that drive Ctx-mediated EGFR internalization were distinct from those found for EGFR degradation mediated by Ctx-M6Pn (fig. S6). Gene ontology (GO) analysis for the hits from either Ctx or LYTAC treatment differentiated the cellular pathways used by these molecules. Enriched sgRNAs from the LYTAC treatment showed cellular components and biological processes involved in endocytosis, whereas sgRNAs from the Ctx treatment used cellular components related to known Ctx-mediated trafficking and nuclear localization of EGFR. We found “retromer complex,” “CUL3-RING ubiquitin ligase,” and “protein neddylation” as enriched specific pathways for LYTAC-mediated EGFR degradation (fig. S7, A and B). Mapping the positive effect hits from LYTAC treatment into subcellular components identified several endolysosomal pathway drivers such as “clathrin-coated pit,” “early endosome,” “late endosome,” and “lysosome,” mirroring the trafficking of CI-M6PR. Because these hits are known regulators of endocytosis, we sought to investigate the genes outside of this category, such as genes distinct from the endolysosomal pathway or genes whose knockout can enhance LYTAC-mediated membrane protein degradation (Fig. 2C). Genes associated with the retromer complex, neddylation pathway, and M6P biosynthesis in the endoplasmic reticulum (ER) and Golgi all had significant effects on LYTAC-mediated degradation but without clear mechanistic bases.

## Disruption of retromer complex genes enhances LYTAC-mediated degradation by reducing recycling from the endosomes to the plasma membrane

**Fig. 3.**
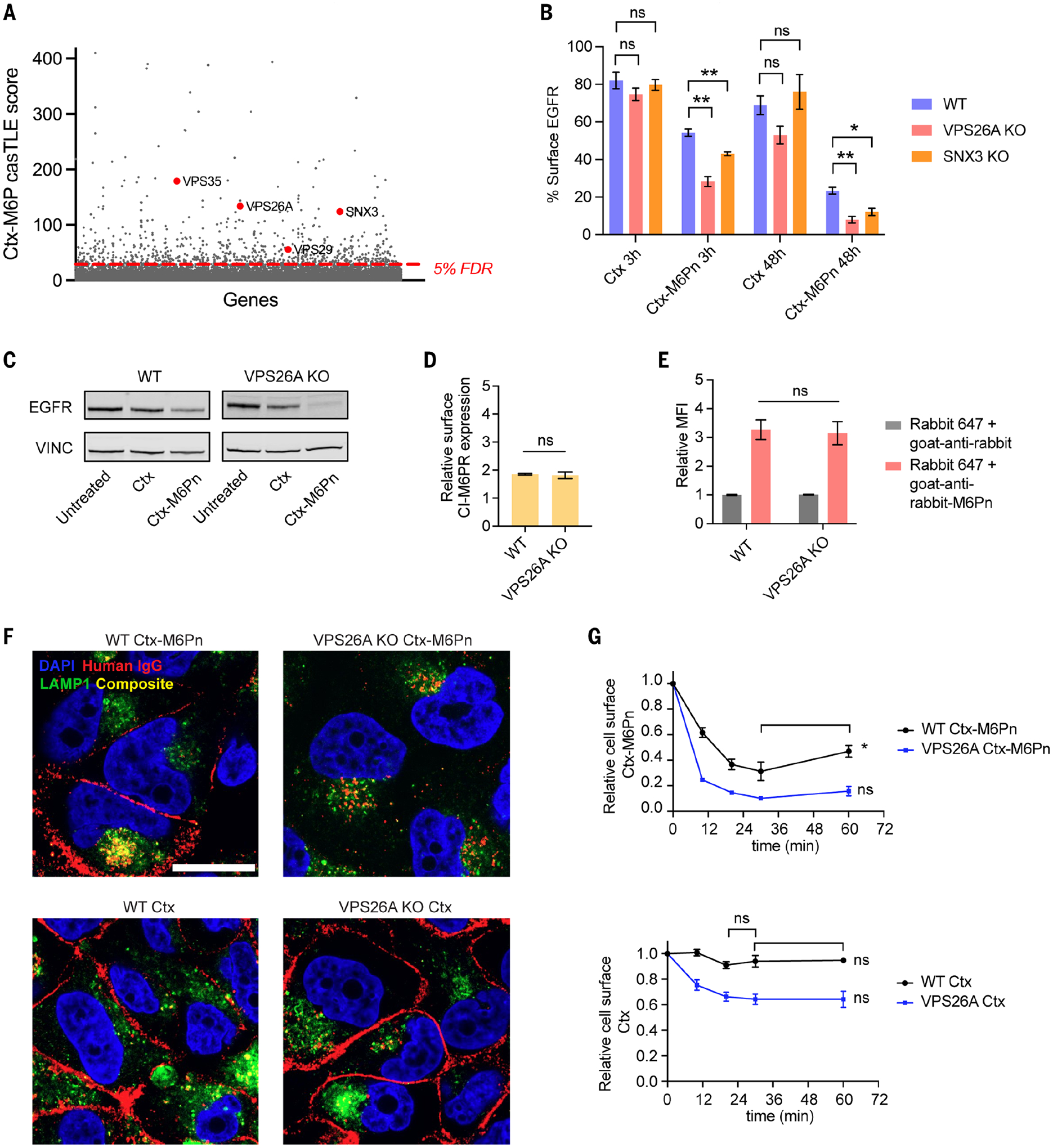
Disrupting retromer complex genes enhances CI-M6PR–mediated target degradation by reducing the recycling of LYTAC–CI-M6PR complexes. (**A**) Retromer complex genes are negative hits from the CRISPR screen. (**B**) Degradation of EGFR in UMRC2 WT, VPS26A KO, and SNX3 KO cells as determined by live-cell flow cytometry after 3 hours or 48 hours of treatment with 10 nM Ctx or Ctx-M6Pn. (**C**) Immunoblot analysis of EGFR levels in WT or VPS26A KO cells after treatment with 10 nM Ctx or Ctx-M6Pn for 48 hours. (**D**) Cell surface expression level of CI-M6PR in WT and VPS26A KO cells by live-cell flow cytometry. (**E**) MFI relative to the control (rabbit IgG-647 only) for WT and VPS26A KO cells incubated at 37°C for 1 hour with 50 nM rabbit IgG-647 and 25 nM goat anti-rabbit or goat anti-rabbit M6Pn. MFI was determined by live-cell flow cytometry. (**F**) Localization of EGFR and Ctx-M6Pn or Ctx (goat anti-human-647) in WT and VPS26A KO cells after pulse treatment of Ctx-M6Pn. Cells were treated with 10 nM Ctx-M6Pn for 24 hours, then washed and incubated with fresh media for additional 24 hours. Scale bar, 10 μm. DAPI, 4′,6-diamidino-2-phenylindole. (**G**) Recycling of Ctx or Ctx-M6Pn determined by live-cell flow cytometry. UMRC2 WT and VPS26A KO cells were pulse treated with 10 nM Ctx or Ctx-M6Pn for 24 hours, then washed and incubated with fresh media for the indicated times followed by surface staining with anti-human-647 on ice. For (B), (D), (E), and (G), data represent three independent experiments and are shown as means ± SEMs. *P* values were determined by unpaired two-tailed *t* tests. NS, not significant; *P ≤ 0.05; **P ≤ 0.01. Data in (C) are representative of three independent experiments. Images in (F) are representative of three independent experiments and are a single plane.

The retromer complex is involved in retrograde transport from endosomes to the trans Golgi network (TGN) or the plasma membrane. Core components of the retromer complex, such as vacuolar protein sorting–associated protein 35 (VPS35), vacuolar protein sorting–associated protein 26A (VPS26A), sorting nexin 3 (SNX3), and vacuolar protein sorting–associated protein 29 (VPS29), were identified as negative hits (Fig. 3A, fig. S8, and table S1) from our screen. The retromer complex is responsible for retrograde transport of CI-M6PR through engagement with sorting signals within the cytosolic tail of CI-M6PR (31–33). Given that retromer genes had negative effect sizes, we questioned whether knockout of these genes would improve LYTAC-mediated degradation. We observed significantly greater degradation of EGFR in VPS26A- or SNX3-deficient cells after 3-hour and 48-hour treatment with LYTAC, which demonstrates that degradation is more efficient when retromer genes are disrupted (Fig. 3, B and C). We achieved >90% degradation of cell surface EGFR in VPS26A-deficient cells. In addition, confocal imaging revealed that EGFR was localized in both late endosomes or lysosomes (LE/LY) and at the plasma membrane of wild-type (WT) cells, whereas EGFR was mostly undetectable on the cell surface of VPS26A-deficient cells after LYTAC treatment (fig. S9A). Loss of VPS26A did not change the surface expression level of CI-M6PR (Fig. 3D). In LYTAC-treated cells, total CI-M6PR levels remained unchanged in both WT and VPS26A-deficient cells, whereas EGFR levels were reduced in both cell types, which confirms that LYTAC degrades its target without degrading CI-M6PR (fig. S9B). Furthermore, no change in LYTAC-mediated internalization of rabbit IgG was observed in VPS26A-deficient cells at 10, 20, and 60 min (Fig. 3E and fig. S9C).

Because the retromer complex is involved in recycling cargo from endosomes, it may partially recycle the LYTAC–CI-M6PR complex before dissociation of LYTAC from the receptor. To test this, we performed a pulse-chase experiment where we treated with Ctx-M6Pn for 24 hours and then washed and incubated with fresh media for an additional 24 hours. The localization of Ctx-M6Pn (LYTAC) was then visualized by goat-anti-human-647 staining. In WT cells, LYTAC was localized at both the plasma membrane and partially in LE/LY, whereas we did not observe LYTAC localization on the cell surface in VPS26A KO cells (Fig. 3F and fig. S10). Using flow cytometry, we observed an increase in cell surface staining of LYTAC with anti-human-647 in WT cells over time, whereas no increase in staining was observed in VPS26A-deficient cells (Fig. 3G). Thus, LYTACs can recycle back to the plasma membrane from endosomes via the retromer complex, whereas such recycling is reduced when a component in the retromer complex is disrupted. To identify whether the recycling of LYTAC was because of recycling of EGFR or CI-M6PR, we conducted the same pulse experiment with Ctx and observed that Ctx was still bound to EGFR on the cell surface in both WT and VPS26A-deficient cells (Fig. 3, F and G). Thus, the retromer complex can recycle the LYTAC–CI-M6PR complex before dissociation of LYTAC from the receptor, and perturbation of retromer genes can increase the efficacy of LYTAC by removing a competitive recycling pathway of CI-M6PR.

## Neddylation of CUL3 is essential and a predictive marker for LYTAC-mediated membrane protein degradation

**Fig. 4.**
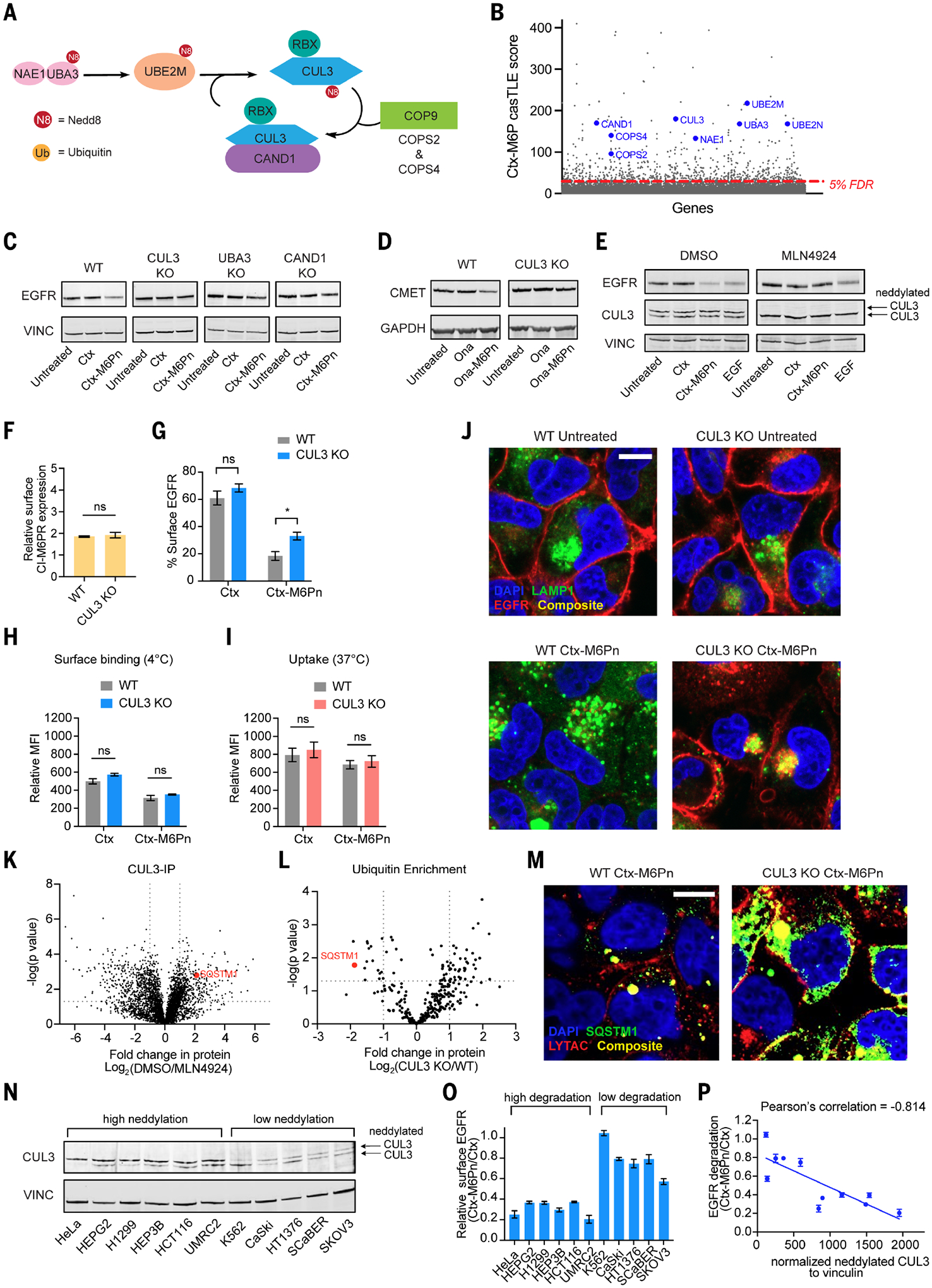
Neddylation of CUL3 is essential for CI-M6PR–mediated target lysosomal degradation. (**A**) CUL3 neddylation process. Neddylation activates E3 ligase, CUL3. (**B**) Genes involved in CUL3 neddylation are positive hits from the CRISPR screen. (**C**) Immunoblot analysis of EGFR levels in WT and CUL3, UBA3, and CAND1 KO cells after treatment with 10 nM Ctx or Ctx-M6Pn for 48 hours. (**D**) Levels of c-MET in WT and CUL3 KO cells after treatment with 10 nM Ona or Ona-M6Pn for 48 hours. (**E**) Immunoblot analysis of EGFR levels in HeLa cells pretreated with DMSO or MLN4924 (2 μM) for 24 hours, then treated with 10 nM Ctx or Ctx-M6Pn for 24 hours. (**F**) Cell surface expression level of CI-M6PR in WT and CUL3 KO cells by live-cell flow cytometry. (**G**) Degradation of cell surface EGFR in WT and CUL3 KO cells as determined by live-cell flow cytometry after 48 hours of treatment with 10 nM Ctx or Ctx-M6Pn. (**H**) Surface staining of Ctx or Ctx-M6Pn with goat-anti-human-647 in WT or CUL3 KO cells measured by live-cell flow cytometry after 1.5-hour incubation on ice. (**I**) Live-cell MFI relative to the control (human IgG-647 only) for WT or CUL3 KO cells incubated at 37°C for 1.5 hours with 50 nM human IgG-647 and 25 nM Ctx or Ctx-M6Pn. (**J**) Visualization of EGFR degradation in WT and CUL3 KO cells using confocal microscopy after continuous treatment with Ctx-M6Pn for 72 hours. Scale bar, 10 μm. (**K**) Volcano plot of interactors after CUL3 immunoprecipitation in cells treated with or without MLN4924 (2 μM). (**L**) Volcano plot of ubiquitin enrichment via quantitative proteomics in WT and CUL3 KO cells. (**M**) Visualization of SQSTM1 and Ctx-M6Pn (IgG) in WT or CUL3 KO cells using confocal microscopy after 48-hour treatment with 10 nM Ctx-M6Pn. Scale bar, 10 μm. (**N**) Immunoblot analysis of CUL3 and neddylated CUL3 across various cell lines. (**O**) Degradation EGFR in various cell lines as determined by relative surface EGFR after 48-hour treatment with 10 nM Ctx-M6Pn versus Ctx by live-cell flow cytometry. (**P**) A scatter plot of EGFR degradation (Fig. 3K) versus normalized neddylated CUL3 expression to vinculin (Fig. 3L) with calculated Pearson’s correlation. For (F), (G), (I), (J), and (O), data represent three independent experiments, and data are shown as means ± SEMs. *P* values were determined by unpaired two-tailed *t* tests. NS, not significant; *P ≤ 0.05. For (C), (D), (E), (M), and (N), data represent three independent experiments. For (H), (J), (M), and (N), data are representative of two independent experiments. Images are a single plane.

We found several genes with high casTLE scores that are involved in neddylation and activation of the E3 ligase cullin-3 (CUL3). During the activation process, Nedd8 is transferred from E1 [Nedd8 activating enzyme E1 subunit 1 (NAE1), ubiquitin-activating enzyme 3 (UBA3)] to E2 [Nedd8-conjugating enzyme Ubc12 (UBE2M)], then to E3 ligase (CUL3). Inactive CUL3 is normally bound by cullin-associated Nedd8-dissociated protein1(CAND1), and upon neddylation, CAND1 dissociates activating CUL3. CUL3 neddylation can be reversed by the constitutive photomorphogenesis 9 (COP9) signalosome complex to allow recycling of neddylation (34–36) (Fig. 4A and fig. S11A). All of these components, including CUL3, were identified as hits with positive effect for Ctx-M6Pn–mediated degradation and were not enriched from the Ctx screen, which suggests that neddylation of CUL3 is important for LYTAC-mediated degradation (Fig. 4B). Knockout of genes (*CUL3*, *UBA3*, *CAND1*) in this pathway (fig. S11, B and C, and table S1) confirmed that degradation of EGFR was dependent on a functional E3 neddylation system (Fig. 4C and fig. S12A). To determine whether a CUL3 dependency was specific to EGFR degradation, we tested for degradation of c-MET in CUL3-deficient cells and found that c-MET degradation was also attenuated (Fig. 4D and fig. S12B), which suggests that the role of CUL3 is not target specific. The impact of neddylation was also examined with MLN4924, which effectively blocked neddylation of CUL3 and completely abrogated LYTAC-mediated degradation (fig. S13A). To verify this effect in a different cell line, we also confirmed that MLN4924 inhibited neddylation of CUL3 and prevented LYTAC-mediated degradation of EGFR and c-MET in HeLa cells. Unlike UMRC2 cells, HeLa cells down-regulate EGFR with EGF treatment. Treatment with MLN4924 had little effect on the EGF-mediated degradation of EGFR, which suggests that CUL3 neddylation is dispensable for EGFR down-regulation by endogenous mechanisms but is essential for LYTAC-mediated degradation (Fig. 4E and fig. S13, B and C). We also determined that recycling of neddylation is essential for LYTAC activity because treatment with a deneddylation inhibitor, CSN5i, abolished LYTAC activity (fig. S14). This is consistent with previous studies that have shown that dynamic neddylation and deneddylation are requirements for active cullin ligases (37, 38).

To determine the stage of the LYTAC-mediated degradation pathway dependent on neddylation of CUL3, we first observed that the cell surface expression of CI-M6PR was unchanged in CUL3-deficient cells (Fig. 4F). We then asked whether CUL3 affects the internalization of LYTAC-target complexes. Although no reduction in total EGFR levels was observed after LYTAC treatment in CUL3-deficient cells (Fig. 4C), we observed a reduction in cell surface EGFR levels relative to the untreated control after LYTAC treatment (Fig. 4G). We confirmed that the binding and internalization of LYTACs did not change in CUL3-deficient cells (Fig. 4, H and I, and fig. S15). Loss of CUL3 also did not affect the LYTAC-mediated internalization of a soluble IgG (fig. S16). Thus, CUL3 activity is downstream of LYTAC-mediated internalization. To further investigate this effect, we looked at the localization of EGFR after 72 hours of LYTAC treatment and found that EGFR was concentrated partially in LE/LY vesicles in CUL3-deficient cells, whereas EGFR was mostly degraded in the WT cells (Fig. 4J). In addition, we performed a pulse-chase experiment where we treated with Ctx or LYTAC for 24 hours and then washed and incubated with fresh media. After 48 hours, LYTAC was cleared in WT cells, as indicated by the lack of anti-human-647 signal. However, LYTAC was observed in puncta that colocalized with CI-M6PR in CUL3-deficient cells (fig. S17), which suggests inefficient transport of LYTAC into lysosomes. Thus, CUL3 plays an essential role in LYTAC-mediated target maturation from endosome to lysosome for degradation.

To understand how CUL3 may regulate maturation of endosomes to lysosomes, we identified interactors of neddylated CUL3 through immunoprecipitation (IP)–proteomics. Because CUL3 is activated by neddylation, we looked for interactors that were depleted when neddylation was inhibited by MLN4924 treatment (Fig. 4K). Substrates that are ubiquitinated by CUL3 were identified by ubiquitin enrichment proteomics in WT and CUL3-deficient cells (Fig. 4L). A prominent hit was sequestosome-1 (SQSTM1) (Fig. 4, K and L). We confirmed enrichment of SQSTM1 after CUL3-IP in the absence of MLN4924 by immunoblot (fig. S18A). SQSTM1 is ubiquitinated by the E3 ligase RNF26, which allows its recruitment of vesicle adaptors involved in endosomal maturation and efficient cargo transfer (39). Our proteomics results suggest that ubiquitination of SQSTM1 by CUL3 is essential for late endosomal maturation and LYTAC activity. We confirmed that LYTACs had reduced ability to promote degradation of EGFR in SQSTM1 KO cells (fig. S18, B to D). Rather, EGFR was trapped in LE/LY compartments after LYTAC treatment (fig. S18E), similarly to CUL3 KO cells. Furthermore, SQSTM1 colocalized with Ctx-M6Pn in CUL3-deficient cells, whereas this behavior was less pronounced in WT cells (Fig. 4M and fig. S18F). Thus, SQSTM1 interacts with LYTAC-containing endosomes but requires ubiquitination at the late endosome by CUL3 to enable further maturation and trafficking of LYTAC-target complexes to lysosomes.

Given the critical role of CUL3 neddylation for LYTAC activity, we questioned whether the expression of neddylated CUL3 could be correlated with degradation efficacy. We compared the expression levels of neddylated CUL3 and EGFR degradation ability across 11 cell lines spanning eight different tissues and observed that cell lines with higher levels of neddylated CUL3 exhibited better degradation of EGFR (Fig. 4, N to P). This correlation suggests that neddylated CUL3 could serve as a predictive marker for LYTAC-mediated membrane protein degradation.

## Disruption of M6P biosynthesis genes enhances LYTAC efficacy

**Fig. 5.**
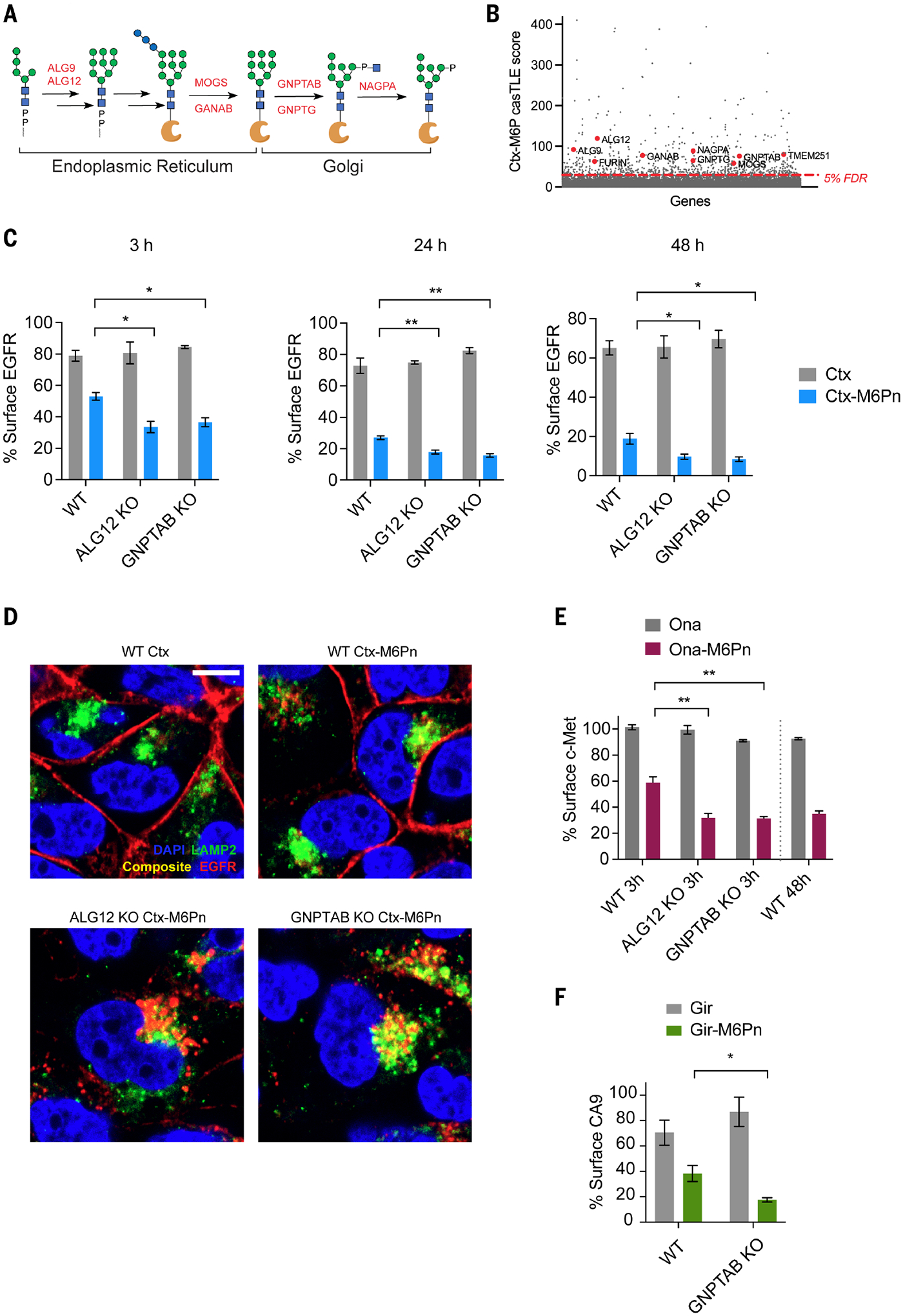
Knockout of M6P biosynthesis genes enhances LYTAC efficacy. (**A**) M6P–N-glycan biosynthesis of lysosomal hydrolases in the ER and Golgi. (**B**) Genes involved in M6P biosynthesis pathway are negative hits from the CRISPR screen. (**C**) Degradation of EGFR in UMRC2 WT, ALG12 KO, and GNPTAB KO cells as determined by live-cell flow cytometry after 3 hours, 24 hours, and 48 hours of treatment with 10 nM Ctx or Ctx-M6Pn. (**D**) Visualization of EGFR degradation in WT and GNPTAB KO cells using confocal microscopy after continuous treatment with Ctx or Ctx-M6Pn for 24 hours. Scale bar, 10 μm. (**E**) Degradation of c-MET in UMRC2 WT, ALG12 KO, and GNPTAB KO cells as determined by live-cell flow cytometry after 3 hours and 48 hours of treatment with 10 nM Ctx or Ctx-M6Pn. (**F**) Degradation of CA-9 in UMRC2 WT and GNPTAB KO cells as determined by live-cell flow cytometry after 24 hours of treatment with 10 nM Ctx or Ctx-M6Pn. For (C), (E), and (F), data represent three independent experiments, and data are shown as means ± SEMs. *P* values were determined by unpaired two-tailed *t* tests. NS, not significant; *P ≤ 0.05; **P ≤ 0.01. Images in (D) are representative of two independent experiments and are a single plane.

Lysosomal hydrolases are synthesized in the ER and transported to the TGN, where they are modified with M6P. CI-M6PR in the TGN recognizes M6P-modified lysosomal proteins and traffics them to late endosomes for eventual lysosomal delivery (40–42). Our screen for LYTAC-mediated degradation identified several glycosyltransferases with negative effect sizes that are involved in the M6P glycoprotein biosynthesis pathway, including alpha-1,2-mannosyltransferase (ALG9), alpha-1,6-mannosyltransferase (ALG12), mannosyl-oligosaccharide glucosidase (MOGS), glucosidase II alpha subunit (GANAB), *N*-acetylglucosamine-1-phosphate transferase (GNPTAB), and *N-*acetylglucosamine-1-phosphodiester alpha-*N-*acetylglucosaminidase (NAGPA) (Fig. 5, A and B; fig. S19A; and table S1). Transmembrane protein 251 (TMEM251) or lysosomal enzyme trafficking factor (LYSET), which was recently characterized as a crucial protein for M6P modification and trafficking of lysosomal hydrolases (43, 44), was also identified (Fig. 5B). Knockouts of ALG12 or GNPTAB resulted in greater lysotracker signal (fig. S19, B to D), consistent with previous accounts of enlarged lysosomes in GNPTAB KO cells (45). Ctx-M6Pn–mediated degradation was enhanced in ALG12- or GNPTAB-deficient cells after 3, 24, and 48 hours of treatment (Fig. 5C). After 48 hours of treatment, we observed >90% depletion of surface EGFR in these cells. By confocal microscopy, some EGFR remained on the cell surface in WT cells after 24 hours of LYTAC treatment, whereas EGFR was nearly depleted from the cell surface in ALG12- or GNPTAB-deficient cells (Fig. 5D). We did not detect any surface-bound EGFR after LYTAC treatment in these cells, indicating enhanced internalization of target-LYTAC–CI-M6PR complexes. We also examined c-MET and CA9 degradation in ALG12 and GNPTAB KO cells. After 3 hours of treatment with Ona-M6Pn, about 40% of surface c-Met was degraded in WT cells. However, we observed 70% degradation of c-Met in ALG12- and GNPTAB-deficient cells, which was the *D*_max_ (maximal percent degradation) reached after 48 hours in WT cells (Fig. 5E). Degradation of CA9 was similarly improved in GNPTAB-deficient cells with Gir-M6Pn, which confirms that disruption of M6P biosynthesis enhances LYTAC activity across multiple membrane targets (Fig. 5F).

## M6P biosynthesis attenuates CI-M6PR cell surface accessibility

**Fig. 6.**
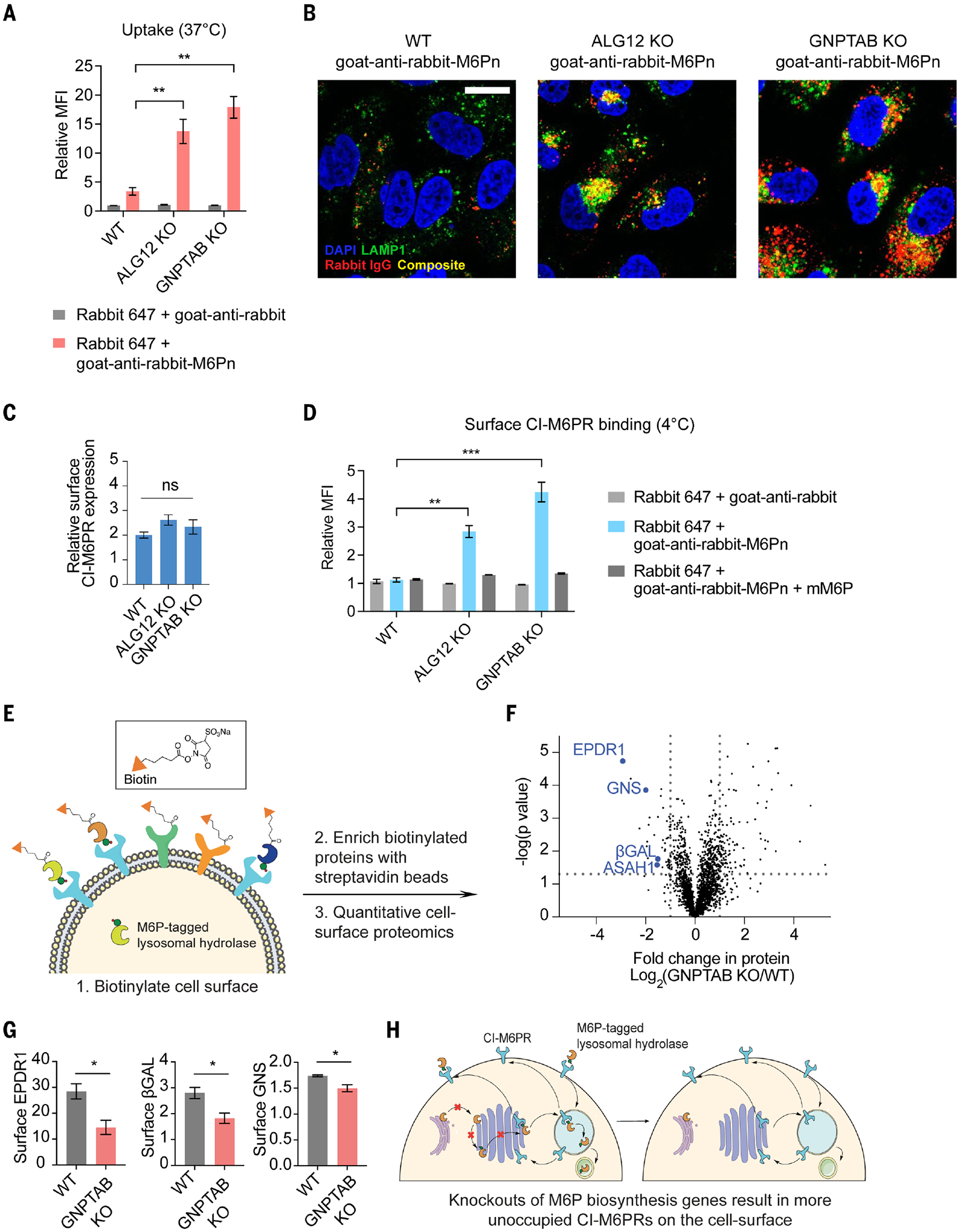
M6P biosynthesis attenuates CI-M6PR cell surface accessibility. (**A**) Uptake of rabbit IgG-647. MFI relative to the control (rabbit IgG-647 only) for WT, ALG12 KO, and GNPTAB KO cells incubated at 37°C for 1 hour with 50 nM rabbit IgG-647 and 25 nM goat anti-rabbit or goat anti-rabbit M6Pn. MFI was determined by live-cell flow cytometry. (**B**) Live-cell imaging of UMRC2 WT, ALG12 KO, and GNPTAB KO cells that were incubated at 37°C for 1 hour with 50 nM rabbit IgG-647 and 25 nM goat anti-rabbit, goat anti-rabbit M6Pn. Scale bar, 20 μm. (**C**) Cell surface expression level of CI-M6PR in WT, ALG12 KO, and GNPTAB KO cells by live-cell flow cytometry. (**D**) Cell surface CI-M6PR binding. MFI relative to the control (rabbit IgG-647 only) for WT, ALG12 KO, and GNPTAB KO cells incubated at 4°C for 30 min with 50 nM rabbit IgG-647 and 25 nM goat anti-rabbit or goat anti-rabbit M6Pn. MFI was determined by live-cell flow cytometry. (**E**) Experimental setup for surface proteomics. WT or GNPTAB KO cells were incubated with NHS-sulfo-biotin on ice for 30 min and were enriched with streptavidin magnetic beads. (**F**) Relative quantitative surface proteomics between WT and GNPTAB KO cells. (**G**) Cell surface expression level of EPDR1, B-gal, and GNS in WT and GNPTAB KO cells. (**H**) Model for knockout of M6P biosynthesis genes resulting in an increased fraction of accessible CI-M6PR on the cell surface. For (A), (C), (D), and (G), data represent three independent experiments, and data are shown as means ± SEMs. *P* values were determined by unpaired two-tailed *t* tests. NS, not significant; *P ≤ 0.05; **P ≤ 0.01; ***P ≤ 0.001. Images in (B) are representative of two independent experiments and are a single plane. For (F), data are representative of three independent experiments.

To elucidate how the disruption of M6P biosynthesis genes improves LYTAC efficacy, we first assessed internalization of soluble cargo at 37°C using rabbit IgG-647. We observed a significant increase in uptake in ALG12- and GNPTAB-deficient cells, where internalized cargo colocalized with lysotracker (Fig. 6, A and B). This may be attributed to increased expression of cell surface CI-M6PR. However, loss of ALG12 or GNPTAB did not result in significant differences in surface CI-M6PR expression (Fig. 6C). Reduced M6P biosynthesis may result in altered CI-M6PR occupancy by lysosomal hydrolases that traffic from the TGN to the plasma membrane, thereby increasing the fraction of unoccupied cell surface CI-M6PR accessible to LYTACs. To test this, we measured the binding of LYTAC to the cell surface by staining with rabbit IgG-647 and goat-anti-rabbit-M6Pn without internalization. Binding of a LYTAC to the cell surface was higher in ALG12- and GNPTAB-deficient cells and was ablated by cotreatment with inhibitory monomeric M6P (mM6P), which indicates that the binding is CI-M6PR dependent (Fig. 6D). Thus, a majority of cell surface CI-M6PR remains occupied at steady state.

We sought to identify proteins occupying cell surface CI-M6PR using quantitative surface proteomics comparing GNPTAB KO cells with WT cells, where we labeled the cell surface with cell-impermeable NHS-sulfo-biotin on ice and enriched cell lysates for biotinylated surface proteins (Fig. 6E). Quantitative proteomics showed a decrease in lysosomal proteins or hydrolases, such as mammalian ependymin-related protein 1 (EPDR1), N-acetylglucosamine-6-sulfatase (GNS), β-galactosidase (β-gal), and acid ceramidase (ASAH1), on the cell surface of GNPTAB-deficient cells compared with the WT cells (Fig. 6F). GNS (46, 47), β-gal (46, 48), and ASAH1 (46, 47) are well-known lysosomal hydrolases that have M6P modifications. EDPR1 is a lysosomal protein with unknown function, but it is modified with M6P (46, 47, 49–51). We confirmed that levels of EPDR1 were also reduced in GNPTAB-deficient cells by immunoblot (fig. S18E), consistent with prior work demonstrating lower cellular levels of M6P-tagged proteins in GNPTAB KO cells (45). To validate our findings from surface proteomics, we analyzed the cell surface expression level of EPDR1, β-gal, and GNS by flow cytometry and confirmed that cell surface levels of these proteins were lower in the GNPTAB-deficient cells (Fig. 6G). Thus, CI-M6PR is occupied with M6P-modified proteins on the cell surface at a steady state. As a result, perturbation of M6P biosynthesis genes increases the fraction of accessible CI-M6PRs on the cell surface, thereby enhancing internalization of LYTACs (Fig. 6H).

## Discussion

First-generation LYTACs could not achieve beyond 70 to 80% target degradation after 24 to 48 hours of treatment, regardless of the specific protein target (7). These findings suggested that unknown aspects of the LYTAC endolysosomal trafficking mechanism may place a limit on protein degradation. We were curious whether this constraint could be overcome through a more nuanced understanding of these trafficking pathways. As with other degradation modalities, we observed that the *D*_max_ values differ across cell lines, even when the same protein is targeted. Discovery of features that drive this variation might allow the prediction of efficacy of LYTAC degradation through a priori genetic or biochemical analyses. In this work, we determined that disruption of retromer genes reduced the recycling of CI-M6PR–LYTAC complexes from endosomes to the plasma membrane and increased lysosomal degradation to >90%. From a therapeutic standpoint, knocking out retromer complexes may not be desirable because retromer complexes are important for conserved cellular endocytic processes (52). However, these results motivate antibody engineering of LYTACs to release their targets at low pH in endosomes, which could allow the target to degrade regardless of LYTAC recycling and could markedly enhance degradation efficacy. In addition, we identified that neddylation of CUL3 is an essential factor for late endosomal maturation and a predictive marker for LYTAC activity.

CI-M6PR–mediated lysosomal transport is the standard ERT approach for several lysosomal storage disorders. Yet, regulators of CI-M6PR internalization ability and trafficking are not fully understood. Recent deorphanization of TMEM251 as an essential factor for lysosomal enzyme transport and M6P trafficking machinery has highlighted the relevance of functional CI-M6PR–mediated transport in disease contexts (43, 44). Through elucidating the mechanistic principles of LYTACs, we show that M6P biosynthesis attenuates CI-M6PR cell surface accessibility, and perturbation of M6P biosynthesis genes increases the fraction of unoccupied receptors on the cell surface. The observation of discrete M6P-tagged proteins that occupy receptors at the cell surface is supported by prior work which has demonstrated that free receptors were replaced with ligand-occupied receptors at the cell surface of cells treated with weak bases or monensin, which inhibit dissociation of receptor-ligand complexes (53). Although direct pharmacological targeting of M6P biosynthesis might adversely affect lysosomal enzyme activities, our findings reveal important design considerations for future LYTACs with enhanced efficacy. One strategy may be to use a higher-affinity M6P-ligand for CI-M6PR that can outcompete the lysosomal hydrolases. Alternatively, usage of orthogonal binding sites on CI-M6PR, such as the IGF2, urokinase receptor (uPAR), and retinoic acid binding sites, might prove more effective (54–57) (41). Beyond LYTACs, delivery applications dependent on CI-M6PR trafficking, including ERTs or macromolecule delivery platforms, could benefit from targeting alternative regions of the internalizing receptor. The guiding principles that result from our studies can be implemented in other therapeutics that benefit from enhanced lysosomal trafficking via CI-M6PR.

The field of extracellular protein degradation is emerging as a powerful method for therapeutic modulation of deleterious proteins. In this work, we used an unbiased functional genomic-driven mechanistic study to characterize the cellular determinants of an extracellular protein degradation platform (LYTACs). The genomic screen approach via protein A magnetic enrichment that we demonstrate can be applied to any other extracellular antibody-based degraders, such as PROTABs, KineTACs, and AbTACs, that harness different lysosome trafficking receptors and pathways. The unbiased screen resulted in fundamental information that sheds light on CI-M6PR occupancy and trafficking. Similar approaches may facilitate identification of the drivers of other degraders and the mechanistic nuances of alternative lysosome trafficking receptors that have been harnessed for degradation, such as ASGPR, RNF43, ZNRF3, and CXCR7 (8–13). Our work thus provides a clear roadmap for studying mechanistic principles of other extracellular degraders and a valuable lens for studying lysosome trafficking receptors.

## Materials and methods

### General chemical synthesis procedures

Reagent-grade chemical reagents were purchased from Carbosynth, Sigma Aldrich, Click Chemistry Tools, and TCI. All chemical reactions were performed in standard, flame-dried glassware capped with rubber septa under an inert atmosphere of nitrogen unless stated otherwise. Stainless steel syringes or cannulae were used to transfer moisture-sensitive liquids. Thin layer chromatography (TLC) was conducted on precoated glass plates covered with 0.2 mm silica gel for monitoring reactions. TLC plates were visualized with UV light or 5% H_2_SO_4_ in MeOH. Reaction mixtures were purified via column chromatography using Biotage SNAP KP-Sil or Ultra C18 cartridges (10 to 100 g) with a Biotage Isolera Prime ACI automated fraction collector. See the supplementary text (Chemical Synthesis Procedures and Characterization) for detailed synthetic protocols and characterization of new compounds.

### Chemical analysis instrumentation

Proton nuclear magnetic resonance (^1^H NMR) spectra were recorded on a Varian 400 spectrometer and proton-decoupled carbon-13 NMR (^13^C {^1^H} NMR) spectra were recorded on a Varian 500 or Varian 400 spectrometer at 25°C. Spectra were reported in parts per million (ppm) downfield of tetramethylsilane and are referenced to the residual resonances of the protium NMR solvent [CD_3_OD: 3.31 (methanol)] and carbon NMR solvent [CD_3_OD: 49.00 (methanol)]. MestReNova (v12.0.3) was used for all chemical NMR analysis. Data are reported as chemical shift, multiplicity (br, broad; s, singlet; d, doublet; t, triplet; q, quartet; quin, quintet; sept, septet; m, multiplet), coupling constants in Hertz (Hz) and integration. High-resolution mass spectrometric data were obtained on a Thermo Exploris 240 Orbitrap mass spectrometer by the Stanford University Mass Spectrometry (SUMS) core facility.

### Cell lines

Adherent cells were cultured in T75 flasks or 15-cm plates at 37°C in a 5% CO_2_ atmosphere. HeLa, HEPG2, HEP3B, HCT116, NCI-H1299, K562, CaSki, HT1376, SCaBER, and SKOV3 cells were obtained from ATCC. UMRC2 was a generous gift from Dr. Erinn Rankin at Stanford University. HeLa, HEPG2, HEP3B, HT1376, and SCaBER were cultured in Dulbecco’s minimum essential medium (DMEM) supplemented with 10% heat-inactivated fetal bovine serum (HI-FBS) and 1% penicillin/streptomycin. NCI-H1299, K562, CaSKi were cultured in RPMI supplemented with 10% HI-FBS and 1% penicillin/streptomycin. HCT116 and SKOV3 were cultured in McCoy 5A supplemented with 10% HI-FBS and 1% penicillin/streptomycin.

### Antibody-M6Pn conjugation

A 2 to 5 mg/ml solution of antibody (goat-anti-rabbit, Ctx, Gir) was buffer exchanged into phosphate-buffered saline (PBS) using a 7K Zeba size-exclusion column according to the manufacturer’s protocol. To the antibody solution was added 50 equiv. of NHS-(PEG)_4_-azide [20 mg ml^–1^ in dimethyl sulfoxide (DMSO), Click Chemistry Tools], and the reaction was incubated overnight at room temperature (RT). The reaction mixture was filtered using a 7K Zeba size-exclusion column to yield the conjugated antibody.

M6Pn_5_ or M65Pn_2_-BCN glycopeptides (100 equiv.) were weighed into an Eppendorf tube, and a 2 mg ml^–1^ solution of antibody-(PEG)_4-_N_3_ was added. The reaction was manually agitated until the mixture was homogeneous. The reaction mixture was allowed to incubate at RT in the dark for 3 days and was filtered using a 7K Zeba size-exclusion column.

### IgG internalization assay by flow cytometry

Cells were plated (75,000 cells per well in a 24-well plate) 1 day before the experiment. For rabbit-IgG internalization, cells were incubated with 250 μl of complete growth media with 50 nM Rabbit IgG-647 and 25 nM goat anti-rabbit, or goat anti-rabbit-M6Pn for 1 hour. For Ctx or LYTAC internalization, cells were incubated with 250 μl of complete growth media with 50 nM goat-anti-human-647 and 25 nM Ctx or 25 nM Ctx-M6Pn. After incubation, cells were washed with PBS three times, lifted with trypsin, then transferred to a 96-well V-bottom plate. The cells were washed three times with PBS + 0.5% bovine serum albumin (BSA) + 5 mM EDTA, then incubated with Sytox Green for 15 min on ice before flow cytometry analysis.

### IgG internalization and colocalization by live-cell imaging

Cells were plated (50,000 cells per well in an 8-well Labtek) a day before the experiment. Cells were incubated with 200 μl of complete growth media with 50 nM Rabbit IgG-647 and 25 nM goat anti-rabbit or goat anti-rabbit-M6Pn for 1 hour. After incubation, 50 nM lysotracker was added and cells were incubated for additional 15 min at 37°C. Cells were then washed with PBS three times, incubated with Hoechst for 5 min, and imaged by confocal microscopy.

### Protein degradation analysis by immunoblot

Cells were plated (75,000 cells per well in a 24-well plate) 1 day before the experiment. Cells were incubated with 250 μl of complete growth media with 10 nM LYTAC or controls for the indicated time. Cells were then washed with PBS three times and lysed with RIPA buffer supplemented with protease inhibitor cocktail (Roche), 0.1% Benzonase (Millipore-Sigma), and PhosStop (Roche) on ice for 30 min. The cell suspension was scraped and transferred to Eppendorf tubes, and spun down at 21,000 *g* for 15 min at 4°C. The supernatant was collected and the protein concentration was determined by BCA assay (Pierce). Equal amounts of lysates were loaded onto 4 to 12% Bis-Tris gel and separated by sodium dodecyl sulfate–polyacrylamide gel electrophoresis (SDS-PAGE). Then, the gel was transferred onto a nitrocellulose membrane and stained with REVERT Total Protein Stain (LI-COR), then blocked with Intercept Blocking Buffer (TBS) (LI-COR) for 1 hour at RT. The membrane was incubated with primary antibody overnight at 4°C, washed three times with TBS-T. Subsequently, the membrane was incubated with secondary antibody for 1 hour at RT and washed three times with TBS-T for visualization with an Odyssey CLx Imager (LI-COR). Image Studio (LI-COR) was used to quantify band intensities.

### Cell surface antigen staining by flow cytometry

Cells were lifted with trypsin for <5 min, quenched with media, and transferred to a 96-well V-bottom plate. Cells were washed three times with PBS + 0.5% BSA + 5 mM EDTA [fluorescence-activated cell sorting (FACS) buffer] and incubated with primary antibody for 30 min on ice. Cells were washed three times with PBS + 0.5% BSA + 5 mM EDTA (FACS buffer) and incubated with secondary antibody for 30 min on ice. After washing three times with FACS buffer, cells were incubated with either Sytox Green, Sytox Red, or Sytox Blue for 15 min on ice. Flow cytometry was performed on either a BD LSR II or MACSQuant Analyzer 10 Flow Cytometer, and FlowJo software was used to gate on single cells and live cells for analysis.

### Cell surface degradation analysis by flow cytometry

Adherent cells were plated (75,000 cells per well in a 24-well plate) 1 day before the experiment. Cells were incubated with 250 ml of complete growth media with 10 nM LYTAC or controls for indicated time. Cells were then washed with PBS three times, lifted with trypsin for <5 min, quenched with media, and transferred to a 96-well V-bottom plate for staining by flow cytometry as shown above.

### Quantitative proteomics for CA9 degradation

#### Sample processing

UMRC2 cells were plated (375,000 cells per well in a 6-well plate) 1 day before the experiment. Cells were incubated with 1500 μl of complete growth media with PBS (control), 10 nM Gir, or 10 nM Gir-M6Pn for 48 hours, with all conditions performed in triplicate. Cells were then washed with PBS and lysed with RIPA buffer supplemented with protease inhibitor cocktail (Roche), 0.1% Benzonase (Millipore-Sigma), and phosphatase inhibitor cocktail (Cell Signaling Technologies) on ice for 30 min. The cells were scraped, transferred to Eppendorf tubes, and spun down at 21,000 *g* for 15 min at 4°C. The supernatant was collected, protein concentration was determined using BCA assay (Pierce), and ~30 μg of total protein from each sample was processed for quantitative bottom-up proteomics using an micro S-trap (ProtiFi) protocol as follows. The concentration of SDS was brought to 5% SDS and disulfide bonds were reduced by heating samples to 95°C for 10 min after addition of 5 mM DTT. After cooling down to RT for 3 min, reduced cysteine residues were alkylated by incubating in 30 mM iodoacetamide (Sigma, A3221) in the dark at RT for 30 min. Samples were then acidified with phosphoric acid at a final concentration of 1.2% and vortexed. Bind/wash buffer [100 mM tetraethylammonium bromide (TEAB) in 90% methanol] was then added to each sample (seven times the final volume after acidification). 150 μl of each sample were loaded onto micro S-trap columns and spun down at 4000 *g* for 20 s. Flow-through was discarded, and the centrifugation step was repeated until all the solution passed through the column. After three washes with 150 μl bind/wash buffer, trypsin (Promega, V5113) in 50 mM TEAB solution was added to the S-trap at a 1:25 protease:protein ratio and incubated at 47°C for 90 min. After trypsinization, peptides were eluted from the S-trap with 40 μl 50 mM TEAB, 40 μl 0.2% formic acid, and 40 μl 50% acetonitrile and 0.2% formic acid by spinning down at 1000 *g* for 60 s for each elution step. All elution steps were collected in the same tube for a give sample and the total peptide mixtures were lyophilized. Peptides were resuspended in 0.2% formic acid, normalized to concentration using a Nanodrop Spectrophotomerter (Thermo Fisher, absorbance at 205 nm), and analyzed by liquid chromatography–tandem mass spectrometry (LC-MS/MS). Before analysis, one microliter of each sample was taken and combined into a pooled sampled that was used to make the chromatogram library discussed below.

#### LC-MS/MS data acquisition

Proteomics data were acquired using a spectrum-library free data-independent acquisition (DIA) approach that relies on gas-phase fractionation (GPF) to generate DIA-only chromatogram libraries (58, 59). Peptides were separated over a 25 cm Aurora Series Gen2 reverse-phase LC column (75 μm inner diameter packed with 1.6 μm FSC C18 particles, Ion Opticks). The mobile phases (A: water with 0.2% formic acid; B: acetonitrile with 0.2% formic acid) were driven and controlled by a Dionex Ultimate 3000 RPLC nano system (Thermo Fisher). An integrated loading pump was used to load peptides onto a trap column (Acclaim PepMap 100 C18, 5 μm particles, 20 mm length, Thermo Fisher) at 5 μl/min, which was put in line with the analytical column 5.5 min into the gradient. The gradient was held at 0% B for the first 6 min of the analysis, followed by an increase from 0% to 5% B from 6 to 6.5 min, and increase from 5 to 22% B from 6.5 to 66.5 min, an increase from 22% to 90% from 66.5 to 71 min, isocratic flow at 90% B from 71 to 75 min, and re-equilibration at 0% B for 15 min for a total analysis time of 90 min per acquisition. Eluted peptides were analyzed on an Orbitrap Fusion Tribrid MS system (Thermo Fisher). Precursors were ionized was ionized with a spray voltage held at +2.2 kV relative to ground, the RF lens was set to 60%, and the inlet capillary temperature was held at 275°C.

Six chromatogram library files were collected through six repeated injections of the pooled sample only. The instrument was configured to acquire 4 m/z precursor isolation window DIA spectra using a staggered isolation window pattern (60) from narrow mass ranges using window placements optimized by Skyline. DIA MS/MS spectra were acquired with an AGC target of 400,000 charges, a maximum injection time of 54 ms, beam-type collisional dissociation (i.e., HCD) with a normalized collision energy of 33, and a resolution of 30,000 at 200 m/z using the Orbitrap as a mass analyzer. The six gas-phase fractionation chromatogram libraries were collected with nominal mass ranges of 400–500 m/z, 500–600 m/z, 600–700 m/z, 700–800 m/z, 800–900 m/z, and 900–1000 m/z. The exact windowing scheme was downloaded from https://bitbucket.org/searleb/encyclopedia/wiki/Home (58) and is available in the supplementary materials. Precursor MS1 spectra were interspersed every 25 scans with an AGC target of 400,000 charges, a maximum injection time of 55 ms, a resolution of 60,000 at 200 m/z using the Orbitrap as a mass analyzer, and a scan range of either 395–505 m/z, 495–605 m/z, 595–705 m/z, 695–805 m/z, 795–905 m/z, or 895–1005 m/z. For quantitative samples (i.e., the non-pooled samples) the instrument was configured to acquire 25 × 16 m/z precursor isolation window DIA spectra covering 385–1015 m/z using a staggered isolation window pattern with window placements optimized by Skyline (windowing scheme downloaded from the same link as above and available in the supplementary materials). DIA spectra were acquired with the same MS/MS settings described above. Precursor MS1 spectra were interspersed every 38 scans with a scan range of 385–1015 m/z, an AGC target of 400,000 charges, a maximum injection time of 55 ms, and a resolution of 60,000 at 200 m/z using the Orbitrap as a mass analyzer. The detailed parameters were recorded (file included in the supplementary materials). To ensure chromate-graphic conditions were consistent between library generation and quantitative sample acquisition, GPF library runs were acquired in the middle of the set of quantitative samples described below, and the quantitative samples were collected in randomized order.

#### Data analysis

Staggered DIA spectra were demultiplexed from raw data into mzML files with 10 ppm accuracy using MSConvert (61) with settings described in Pino *et al*. (58). Encyclopedia (version 1.12.31) (59) was used to search demultiplexed mzML files using an internal PECAN fasta search engine called Walnut (62) and a reviewed human proteome database (canonical sequences only) from Uniprot (63). Walnut settings were: fixed cysteine carbami-domethylation, full tryptic digestion with up to 2 missed cleavages, HCD (y-only) fragmentation, 10 ppm precursor and fragment mass tolerances, and 5 quantitative ions. The chromatogram library resulting from the Walnut search was then used for Encyclopedia searching, where all analogous settings to the Walnut search remained the same, and other settings included a library mass tolerance of 10 ppm, inclusion of both b- and y-type fragment ions, and a minimum number of quantitative ions set at 3. Percolator (version 3.1) was used to filter peptides to a 1% false discovery rate (FDR) using the target/decoy approach and proteins to a 1% protein-level FDR assuming protein grouping parsimony. Resulting data from EncyclopeDIA were checked in Skyline (64) before further processing with Perseus (65), including filtering so that only proteins that were detected in all replicates of all three conditions were retained and significance calculations for comparisons of Gir and Gir-M6Pn treatments relative to control were performed using a two-tailed *t* test with a permutation-based FDR with 250 randomizations, an FDR of 0.05, and an S0 value of 1.

### Genome-wide CRISPR-Cas9 screen in UMRC2 cells

UMRC2 cells were transduced with LentiArray Cas9 Lentivirus (Invitrogen A32064) and single-cell sorted to obtain stable UMRC2-Cas9 cells. A genome-wide, 10 sgRNA per gene CRISPR deletion library from the Bassik laboratory at Stanford University (29) was synthesized, cloned, and infected into Cas9-expressing UMRC2 cells. Briefly, ~400 million UMRC2-Cas9 cells (4 million cells per 15-cm plate) were infected with the CRISPR KO library at a multiplicity of infection (MOI) of 0.3 to 0.4. Cells expressing sgRNAs were selected for using puromycin (12 μg/ml) for 5 days. Selected cells were then plated for treatments in puromycin-free media.

For the screen, cells were split into two conditions, each in duplicate: Ctx (10 nM) treated group and Ctx-M6Pn (10 nM) treated group. For each replicate and condition, 100 million cells were plated and treated with Ctx or Ctx-M6Pn for 48 hours. Cells were washed with PBS and harvested (~200 million cells per replicate). Cells were resuspended in FACS buffer (0.5% BSA + 2 mM EDTA in PBS) then split into 15 ml falcon tubes with maximum 10 ml per tube (4 million cells/ml). Pierce Protein A Dynabeads were added to the cells (15 μl per 2 million cells) and incubated with rotation at 4°C for 30 min. After incubation, the tubes were placed on a magnetic rack for 2 min. The unbound supernatant was transferred to a new tube (unbound fraction), placed on the magnetic rack again for 2 min to remove any remaining beads, and the supernatant was pelleted and saved. The beads were washed twice with FACS buffer, and the remaining beads were saved and frozen as the bound fraction.

Genomic DNA of each condition was extracted using Qiagen DNA Blood Maxi kit (catalog no. 51194). The sgRNA sequences were amplified and prepared for sequencing as previously described (29). These libraries were then sequenced using an Illumina NextSeq with ~40 million reads per condition. Analysis and comparison of guide composition of Ctx-M6Pn treated versus Ctx treated conditions were performed using casTLE as previously described (30).

### Generation and analysis of knockout cell lines

For all genes except *CUL3* and *SNX3*, UMRC2 cells were nucleofected with a Lonza 4D-Nucleofector Unit using the Lonza SE Cell Line 4D-Nucleofector Kit. sgRNAs and spCas9 were obtained from Synthego’s Gene Knockout Kit v2, and nucleofection was carried out using manufacturer’s protocol. For *CUL3* or *SNX3*, sgRNA targeting the gene was cloned into the lentiCRISPR v2 vector (Addgene, no. 52961) and lentivirus was generated in HEK293T cells and transduced into UMRC2 cells. Cells were subsequently selected with puromycin (12 μg/ml) for two passages.

DNA was extracted using QuickExtract DNA Extraction Solution (Epicentre, catalog no. QE09050) and polymerase chain reaction (PCR) amplification was performed according to Synthego’s “Genotyping” protocol. Knockout analysis was performed using Synthego’s online tool, Inference of CRISPR Edits (ICE), according to the manufacturer’s protocol.

sgRNA CUL3: GAATGATCATCAAACAGCTA

sgRNA UBA3: ACGUUUCUCACUUCAGUGCU, GCUCGAGGAACUUCUUUACA, GCAGAAUGGCUGUUGAUGGU

sgRNA CAND1: GUGAUGAUGAUGACAUGAGU, UGGAUGCUGUAGUUAGCACA, AGAGCGUGAAGAGAAUGUAA

sgRNA VPS26A: UUAUUUCAGAGUUUUCUUGG, UGAUGGGGAAACCAGGAAAA, CGGAGAAUCCGUUUCAGGAA

sgRNA SNX3: GGGGTCCGTAGGCGTCATTC sgRNA ALG12: AGCGUAGCUCCUUGUGUGGC, GACGCACGCGCCGACGGUGC, GCAGCCCAGGCCGCGGGGCA

sgRNA GNPTAB: AGUGACAAUAGUAACACACC, AUGAAAAUAUUCCGAACCCA, UUGAAGAUAACGAAGAACUG

sgRNA SQSTM1: UAUGGCGUCGCUCACCGUGA, CUGCAGCCCCGAGCCUGAGG, CUGCGAGCGGCUGCUGAGCC

### Confocal microscopy for membrane protein degradation

Adherent cells were plated (50,000 cells per well in an 8-well chamber slide) 1 day before the experiment. Cells were incubated with 200 μl of complete growth media with 10 nM LYTAC or controls for indicated time. Cells were then washed with PBS and fixed with 4% paraformaldehyde in PBS for 15 min at RT, washed three times, and permeabilized with 0.1% Triton for 5 min at RT. Cells were blocked in 10% goat serum in PBS for 1 hour at RT, and incubated with primary antibody overnight at 4°C. Cells were washed with DPBS, then incubated with secondary antibody and DAPI for 1 hour at RT. Cells were washed with DPBS and imaged with Nikon A1R confocal microscope using Plan Fluor 60x oil immersion 1.30-numerical aperture objective. 405-nm violet laser, 488-nm blue laser, 561-nm green laser, and 639-nm red laser were used. Single focal plane images were captured and are represented in figures.

### Neddylation inhibition of CUL3

Adherent cells were plated (50,000 to 75,000 cells per well in a 24-well plate) 1 day before the experiment. Cells were pretreated with 2 μM MLN4924 for 24 hours. On the following day, cells were treated with fresh 2 μM MLN4924 and 10 nM Ctx or Ctx-M6Pn for another 24 hours. Cells were then harvested for immunoblot analysis as mentioned above.

### Cell surface binding analysis by flow cytometry

Cells were trypsinized, quenched with media, and transferred to a 96-well V-bottom plate. Cells were washed three times with PBS + 0.5% BSA + 5 mM EDTA (FACS buffer) and incubated with 50 nM Rabbit IgG-647 and 25 nM goat anti-rabbit or goat anti-rabbit-M6Pn for 30 min on ice. Cells were washed three times with PBS + 0.5% BSA + 5 mM EDTA (FACS buffer) and incubated with Sytox Green. Flow cytometry was performed on either a BD LSR II or MACSQuant Analyzer 10 Flow Cytometer, and FlowJo software was used to gate on single cells and live cells for analysis.

### CUL3-IP proteomics

UMRC2 cells were plated 1 day before the experiment (375,000 cells per well in a 6-well plate, 3 wells were used per condition per replicate). Cells were pretreated with 2 μM MLN4924 for 24 hours. On the following day, cells were treated with fresh 2 μM MLN4924 and 10 nM Ctx or Ctx-M6Pn for another 24 hours. Cells were then washed with PBS three times and lysed with RIPA buffer supplemented with protease inhibitor cocktail (Roche), 0.1% Benzonase (Millipore-Sigma), and PhosStop (Roche) on ice for 30 min (100 μl of lysis buffer per 1 well of 6-well plate). Lysates were spun down at 21,000 *g* for 15 min at 4°C. The supernatant was collected and the protein concentration was determined by BCA assay (Pierce). Pierce Protein A Dynabeads were precomplexed with rabbit anti-CUL3 antibody (Novus) (4 μl of antibody in 40 μl of Dynabeads per sample) for 2 hours rotating at RT. Equal amounts of lysates were incubated with antibody-bead complex rotating at 4°C overnight. The next day, the beads were washed three times with RIPA lysis buffer and eluted by boiling at 95°C for 10 min with 5% SDS with 20 mM DTT. The quantitative proteomics workflow for CUL3-IP proteomics was similar to the methods used to investigate CA9 degradation as described above. Samples were digested with trypsin using the micro S-trap protocol, peptide abundance was measured with a Nanodrop Spectrophotomerter (absorbance at 205 nm), and one microliter of each sample combined into a pooled sample that was used to make a chromatogram library for CUL3-IP samples. The same DIA approach and LC-MS/MS data acquisition strategy was used as described above. Data analysis was similar, but instead of EncyclopeDIA, demultiplexed mzML files were processed with FragPipe using the MSFragger-DIA and DIA-NN (66, 67) for quantitative analysis with default settings. Data were further processed with Perseus as described above: protein intensity values were log_2_ transformed before further analysis, proteins with measured values for all three replicates with at least one condition were retained, and missing values were imputed from a normal distribution with a width of 0.3 and a downshift value of 2.5. Significance calculations for pairwise comparisons were performed using a two-tailed *t* test with a permutation-based FDR with 250 randomizations, an FDR of 0.05, and an S0 value of 1.

### Ubiquitin-enrichment proteomics

#### Sample preparation

UMRC2 cells were plated 1 day before the experiment (15-cm plate). The cells were treated with 10 nM Ctx or Ctx-M6Pn for 48 hours. Cells were then washed with PBS three times and lysed with RIPA buffer supplemented with protease inhibitor cocktail (Roche), 0.1% Benzonase (Millipore-Sigma), and PhosStop (Roche) on ice for 30 min (100 μl of lysis buffer per 1 well of 6-well plate). Lysates were spun down at 21,000 *g* for 15 min at 4°C. The supernatant was collected, and the protein concentration was determined by BCA assay (Pierce).

Samples were processed with an S-trap protocol as described above, except modified for the midi columns to digest 5 mg of lysate (as measured by BCA) per condition. After digestion, 10 μg of peptides were kept for quantitative proteomics with DIA methods described above, whereas the rest of the peptides were processed for ubiquitin site profiling with TMT labeling using methods adapted from previous work (68–70). The PTM-Scan ubiquitin remnant motif (K-ɛ-GG) kit (Cell Signaling Technology, kit no. 5562) was used to enrich for the ubiquitin remnant motif. For all steps in the enrichment of K-ɛ-GG peptides except peptide elution, microcentrifuge tubes containing the sample and the antibody beads are kept on ice when not spinning. First, antibody-bound beads were washed three times with 100 mM sodium borate (pH 9.0) and incubated with 20 mM DMP for 60 min at RT. Beads were washed twice with 200 mM ethanolamine and incubated overnight at 4°C with 200 mM ethanolamine. After this incubation, beads were washed three times with immunoprecipitation (IAP) buffer (50 mM MOPS, pH 7.2, 10 mM sodium phosphate, 50 mM NaCl). For each enrichment, 156 μg of crosslinked anti-K-ɛ-GG antibody beads was used. Peptides from each sample were resuspended in 1 mL of ice-cold IAP buffer and vortexed. The pH of each sample was checked by spotting ~1 μL of solution on pH paper to ensure the pH was ~7. Peptide samples were then centrifuged at 20,000 *g* for 5 min to remove any insoluble material. Peptides were added to a tube of aliquoted antibody and incubated for 2 hours at 4°C, with gentle end-over-end rotation. After incubation, all samples were centrifuged at 2000 *g* for 1 min and then antibody beads were allowed to settle by letting tubes sit for ~10 to 20 s on ice. The supernatant (IP flowthrough) was removed. This IP flowthrough was immediately transferred to another aliquot of crosslinked anti-K-ɛ-GG antibody beads for a second enrichment, repeated as described above, meaning each lysate was immunoprecipitated twice. During the 2-hour incubation of the second IP, antibody beads from the first IP were washed with all washes completed as quickly as possible. For washing the beads, after the addition of the wash reagent, the tubes were inverted about five times, centrifuged for 30 s at 2000 *g*, allowed to sit for 10 to 20 s to let the beads settle, and the supernatant was removed. Immediately after removal of the IP flowthrough, two washes with 1 mL ice cold IAP buffer were followed by three washes with 1 μL ice-cold PBS. DiGly peptides were eluted twice with 50 μL 0.15% TFA, by first gently tapping the tube after addition of TFA and letting them set at RT for 5 min. Beads with TFA were then centrifuged at 2000 *g* for 1 min, beads were allowed to settle for 10 s, and supernatants containing the eluted peptides were removed and pooled. This washing and elution process was repeated for the second IP, and elutions from the first and second IP were combined before desalting with Strata-X reversed phase cartridges and drying with vacuum centrifugation. After enrichment, diGly peptides were labeled with TMTpro 16-plexkit (71) according to manufacturer’s instructions (Thermo Fisher Scientific). TMT reagents were equilibrated to RT and resuspended in 20 μL anhydrous ACN. DiGly peptides were resuspended in 20 μL 100 mM TEAB and combined with appropriate TMT labels for a 1-hour incubation at RT. After this, 5 μL of 5% hydroxylamine was added to each sample with a 15-min incubation at RT. TMT-labeled samples were then combined and dried with vacuum centrifugation. Combined samples were then fractionated with a high pH reversed phase spin column kit according to manufacturer’s instructions (Thermo Fisher Scientific) with slight modifications. Six fractions were collected with elution buffers containing 17.5%, 20%, 22.5%, 25%, 30%, and 70% ACN, respectively, made with 0.1% triethylamine as the aqueous fraction. Spin columns were washed once with 300 μL ACN and twice with 0.1% TFA by spinning at 5000 *g* for 2 min. TMT-labeled diGly peptides were resuspended in 0.1% TFA and added to the column with centrifugation for 2 min. The columns were then washed with 300 μL 5% ACN in 0.1% TFA and 300 μL water before collecting six fractions using the elution buffers with increasing ACN percentage described above. Fractions were dried with vacuum centrifugation and resuspended in 0.2% formic acid for LC-MS/MS analysis. TMT labeling conditions are included in table S2.

### LC-MS/MS data acquisition

Nonlabeled, nonenriched peptides from these samples were processed with the same DIA methods described above. TMT-labeled diGly peptides were separated over a 25 cm Aurora Series Gen2 reverse-phase LC column (75 μm inner diameter packed with 1.6 μm FSC C18 particles, Ion Opticks). The mobile phases (A: water with 0.2% formic acid; B: acetonitrile with 0.2% formic acid) were driven and controlled by a Dionex Ultimate 3000 RPLC nano system (Thermo Fisher). An integrated loading pump was used to load peptides onto a trap column (Acclaim PepMap 100 C18, 5 μm particles, 20 mm length, Thermo Fisher) at 5 μl/min, which was put in line with the analytical column 5.5 min into the gradient. The gradient was held at 0% B for the first 6 min of the analysis, followed by an increase from 0% to 5% B from 6 to 6.5 min, and increase from 5 to 25% B from 6.5 to 200 min, an increase from 25% to 90% from 200 to 218 min, isocratic flow at 90% B from 218 to 224 min, and re-equilibration at 0% B for 16 min for a total analysis time of 240 min per acquisition. Eluted peptides were analyzed on an Orbitrap Fusion Tribrid MS system (Thermo Fisher). Precursors were ionized was ionized with a spray voltage held at +2.2 kV relative to ground, the RF lens was set to 60%, and the inlet capillary temperature was held at 275°C. Survey scans of peptide precursors were collected in the Orbitrap from 400 to 1600 Th with an AGC target of 400,000, a maximum injection time of 50 ms, and a resolution of 120,000 at 200 m/z. Monoisotopic precursor selection was enabled for peptide isotopic distributions, precursors of z = 2 to 6 were selected for data-dependent MS/MS scans for 3 s of cycle time, and dynamic exclusion was set to 120 s with a ±10 ppm window set around the precursor monoisotope. An isolation window of 0.7 m/z was used to select precursor ions with the quadrupole, and peptides were analyzed using a synchronous precursor selection (SPS) MS3 method (72). Precursors were fragmented by CID at a normalized collision energy (NCE) of 35%. After acquisition of each MS2 spectrum, an SPS-MS3 scan was collected on the top 10 most intense ions in the MS2 spectrum. SPS-MS3 precursors were fragmented by high-energy collision–induced dissociation (HCD) and analyzed using the Orbitrap (NCE = 50%, AGC = 100,000, maximum injection time = 118 ms, and resolution = 60K).

#### Data analysis

Whole proteome DIA samples were analyzed as described above. Data from the TMT diGly peptides were searched using searched with the Andromeda search engine in MaxQuant (73, 74) using the entire human proteome downloaded from Uniprot9 (reviewed, 20,428 entries). Quantitation settings for “Reporter ion MS3” with TMT-16plex reporter ion masses were used with a reporter ion mass tolerance set to 0.003. Cleavage specificity was set to Trypsin/P with 3 missed cleavages, and variable modifications included the diGly residue on lysine, oxidation of methionine, and acetylation of the protein N terminus with five maximum modifications per peptide. Defaults were used for the remaining settings, including PSM and protein FDR thresholds of 0.01 and 20 ppm, 4.5 ppm, and 0.5 Da for first search MS1 tolerance, main search MS1 tolerance, and MS2 product ion tolerance, respectively. Quantified diGly sites were then processed in Perseus. Contaminants and reverse hits were removed, results were filtered for diGly that had localization probabilities > 0.75, and signal in all relevant TMT channels was required. Significance testing was performed using a two-tailed *t* test with 250 randomizations and an FDR of 0.05.

### Quantitative cell surface proteomics

UMRC2 WT cells or GNPTAB-deficient cells were plated in triplicate in 15-cm plates (10 million cells each) 1 day before the experiment. Cells were washed with cold PBS and incubated in PBS (no biotin control) or 1 mg/ml solution of NHS-sulfo-biotin in PBS with gentle rocking for 30 min at 4°C. Biotin labeling was quenched with 100 mM Tris-HCl, and the cells were washed three times with cold PBS. Cells were scraped using cold PBS and were pelleted and lysed with RIPA buffer supplemented with protease inhibitor cocktail (Roche), 0.1% Benzonase (Millipore-Sigma), and phosphatase inhibitor cocktail (Cell Signaling Technologies) on ice for 30 min. Cells were then clarified by centrifugation at 21,000 *g* for 15 min at 4°C. The supernatant was collected, and the protein concentration was determined by BCA assay (Pierce). Equal amounts of lysates were then incubated with 500 μl of Streptavidin Dynabeads (Pierce) and rotated overnight at 4°C. For enrichment, the beads were pelleted using a magnetic rack and the supernatant was discarded. Beads were washed twice with cold RIPA lysis buffer, once with 1M KCl, once with 0.1M sodium bicarbonate, once with 2M urea in 10 mM Tris-HCl pH 8.0, then twice with RIPA lysis buffer. Biotinylated proteins were eluted from the beads by boiling each sample in 5% SDS supplemented with 2 mM biotin and 20 mM DTT for 10 min. The beads were pelleted, and the eluates were collected and processed using the same S-trap protocol and DIA methods described above. For quantitative comparisons of cell surface protein difference, protein intensity values were log_2_ transformed before further analysis in Perseus, proteins with measured values for all three replicates with at least one condition were retained, and missing values were imputed from a normal distribution with a width of 0.3 and a downshift value of 1.8. Significance calculations for pairwise comparisons were performed using a two-tailed *t* test with a permutation-based FDR with 250 randomizations, an FDR of 0.05, and an S0 value of 1. GO keywords and cellular component terms were used to annotate proteins as lysosomal and cell surface.

### Figure illustration

Parts of Fig. 1 and Fig. 6 were modified from Servier Medical Art, licensed under a Creative Common Attribution 3.0 Generic License.

## Supplementary Material

Supplementary text and figures

supplementary checklist

Data

## Data Availability

All data are available in the manuscript or the supplementary materials. In addition, the mass spectrometry proteomics data have been deposited to the ProteomeXchange Consortium via the PRIDE partner repository with the dataset identifier PXD037899. Inquiries for materials in this manuscript can be directed to the corresponding authors.
