# Supplementary material for: Elucidating the cellular determinants of targeted membrane protein degradation by lysosome-targeting chimeras
Source: Science. Author manuscript; Available in PMC 2024 Jan 4. (PMC10766146; doi:10.1126/science.adf6249)
Supplement: Supplementary text and figures [file NIHMS1952705-supplement-Supplementary_text_and_figures.pdf]

Science AAAS

# Supplementary Materials for

## Elucidating the cellular determinants of targeted membrane protein degradation by lysosome-targeting chimeras

Green Ahn *et al*.

Corresponding authors: Steven M. Banik, sbanik@stanford.edu; Carolyn R. Bertozzi, bertozzi@stanford.edu

*Science* **382**, eadf6249 (2023)
DOI: 10.1126/science.adf6249

**The PDF file includes:**

Supplementary Text
Figs. S1 to 19
Tables S1 to S3

**Other Supplementary Material for this manuscript includes the following:**

MDAR Reproducibility Checklist
Data S1 to S8

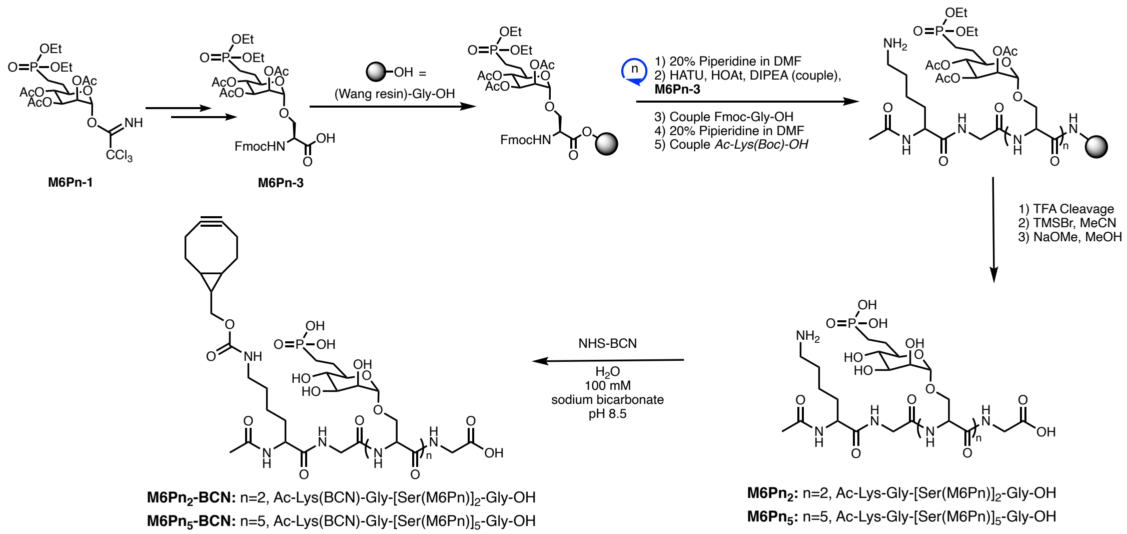


**Fig. S1. Synthesis of M6Pn peptides via solid phase peptide synthesis (SPPS).** Synthetic scheme for $M6Pn_2$ and $M6Pn_5$ peptides.

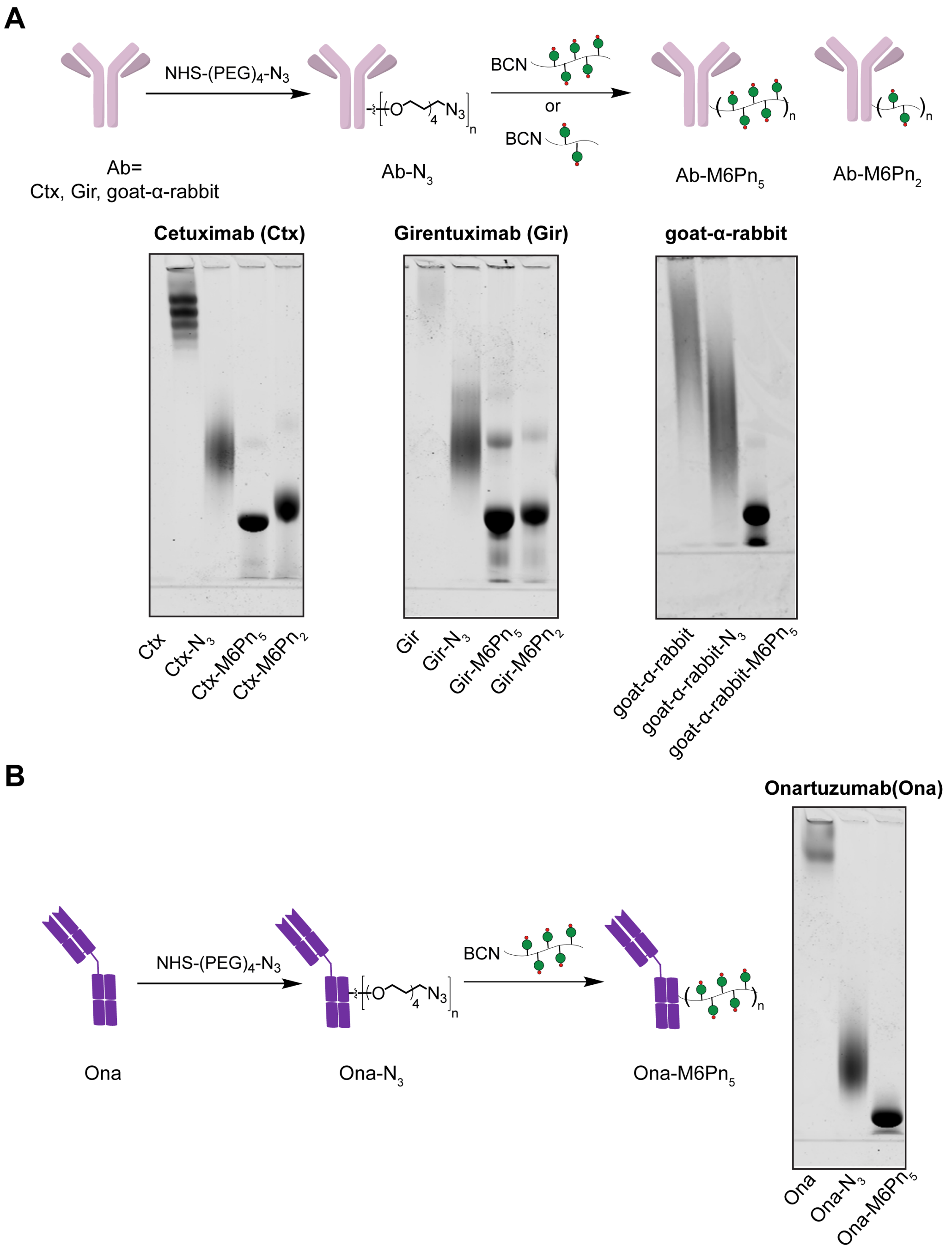


**Fig. S2. Antibody-LYTAC conjugation using lysine functionalization and click chemistry. (A)** Antibodies were reacted with NHS-(PEG)$_4$-N$_3$ followed by BCN-M6Pn$_5$ or BCN-M6Pn$_2$ to yield LYTAC conjugates. Native gel electrophoresis was used to monitor conversion of Ab to Ab-N$_3$, Ab-M6Pn$_5$, and Ab-M6Pn$_2$. **(B)** A single-armed antibody against c-MET (Onartuzumab) was reacted with NHS-(PEG)$_4$-N$_3$ then with BCN-M6Pn$_2$ or BCN-M6Pn$_5$ to yield LYTAC conjugates. Native gel electrophoresis was used to monitor conversion of Ona to Ona-N$_3$ and Ona-M6Pn$_5$.

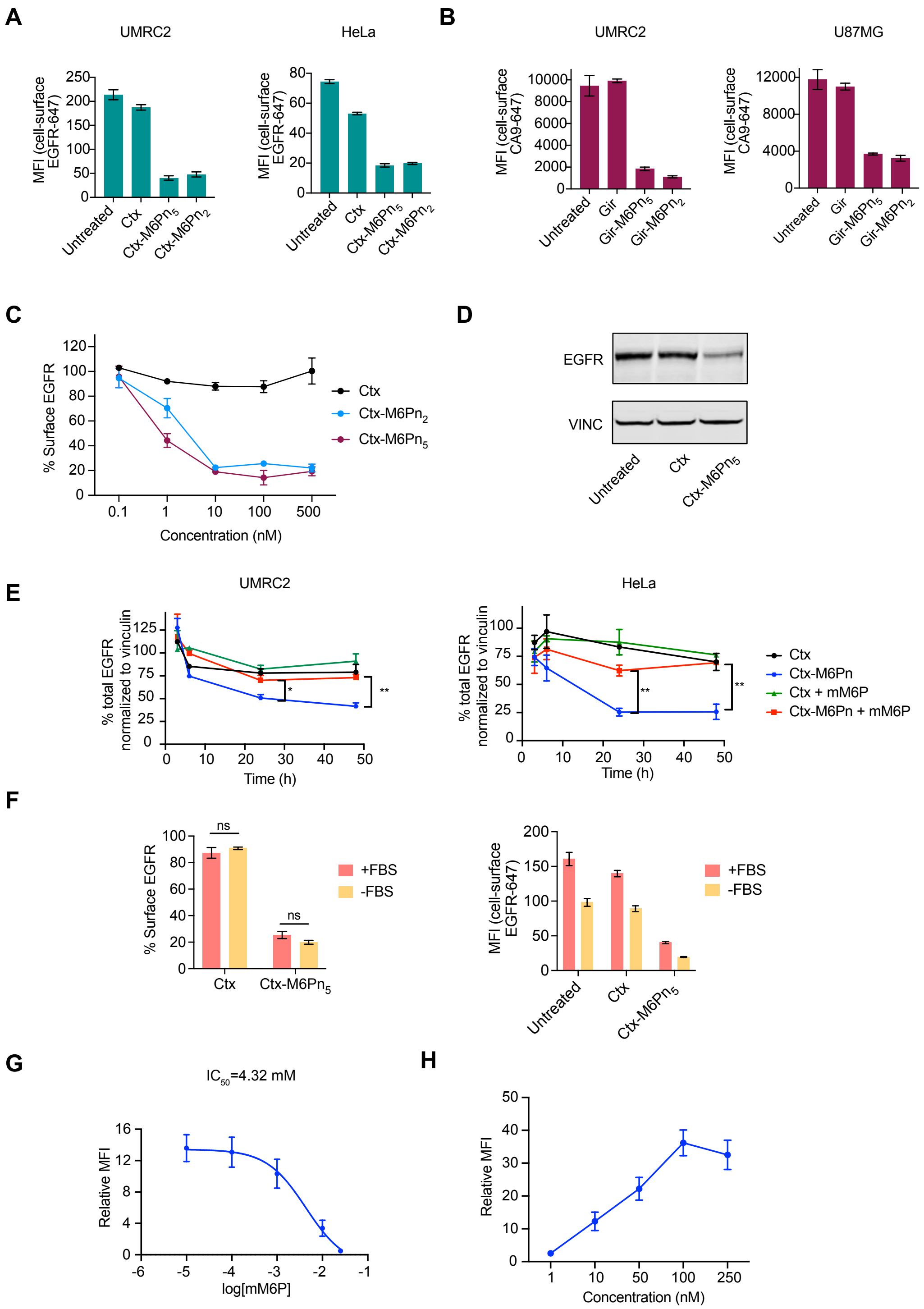


**Fig. S3**. **Characterization of antibody-glycopeptide LYTACs. (A)** Raw mean fluorescence intensity (MFI) for EGFR degradation in Fig. 1C. **(B)** Raw MFI for CA9 degradation in Fig. 1D. **(C)** Dose-dependent degradation of EGFR in UMRC2 cells following 48 h treatment with Ctx, Ctx-M6Pn$_2$, or Ctx-M6Pn$_5$ as determined by live cell flow cytometry. **(D)** Immunoblot analysis of EGFR degradation in UMRC2 cells treated with 10 nM Ctx or Ctx-M6Pn$_5$ for 48h. **(E)** Time-course degradation of EGFR in UMRC2 cells (left) or HeLa cells (right) as determined by

immunoblot quantification of EGFR normalized to vinculin following treatment with 10 nM Ctx or Ctx-M6Pn$_5$ with or without monomeric mM6P inhibition (15 mM). **(F)** Degradation of cell-surface EGFR in media supplemented with or without FBS in UMRC2 cells as determined by live cell flow cytometry following 48 h treatment with 10 nM Ctx or Ctx-M6Pn$_5$. **(G)** IC$_{50}$ of monomeric M6P (mM6P) in LYTAC-mediated uptake. UMRC2 cells were treated with 25 nM of rabbit-647 and 25 nM of goat-anti-rabbit or goat-anti-rabbit-M6Pn$_5$ with varying concentrations of mM6P for 2 h, and cellular uptake was analyzed by live cell flow cytometry. **(H)** Saturation curve of LYTAC-mediated internalization. UMRC2 cells were treated with varying concentrations of rabbit-647 and goat-anti-rabbit or goat-anti-rabbit-M6Pn$_5$ for 2 h, and cellular uptake was analyzed by live cell flow cytometry. All data represent three independent experiments, and data are shown as mean ± S.E.M. P values were determined by unpaired two-tailed t-tests.

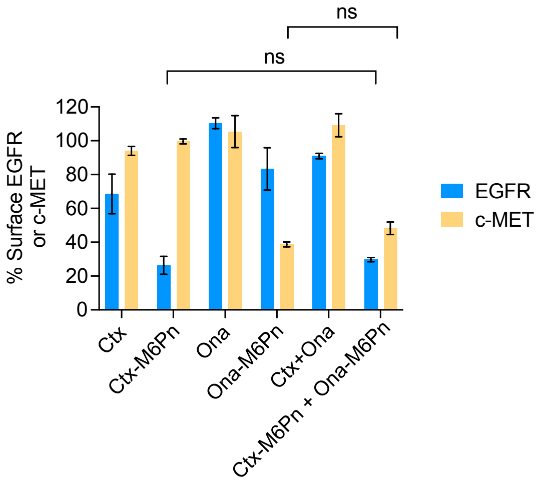


**Fig. S4**. **Simultaneous degradation of EGFR and c-Met.** Depletion of cell surface c-Met and EGFR in HEP3B cells as determined by live-cell flow cytometry following 48 h of single treatment with 10 nM of Ctx or Ona-M6Pn or co-treatment of Ctx and Ona-M6Pn. Data represent three independent experiments, and data are shown as mean ± S.E.M. P values were determined by unpaired two-tailed t-tests.

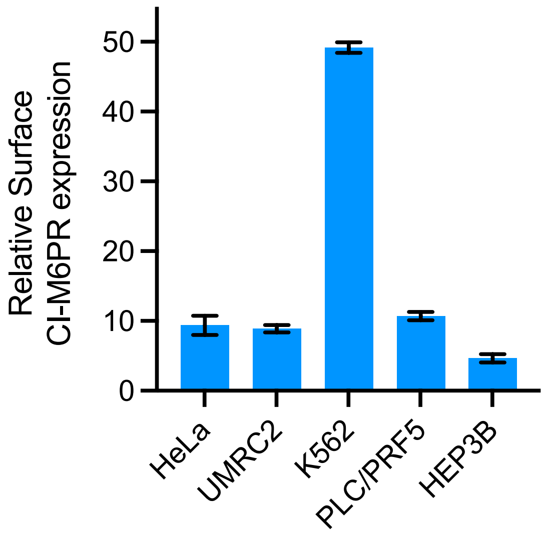


**Fig. S5**. **Cell-surface CI-M6PR expression across different cell lines by live-cell flow cytometry.**

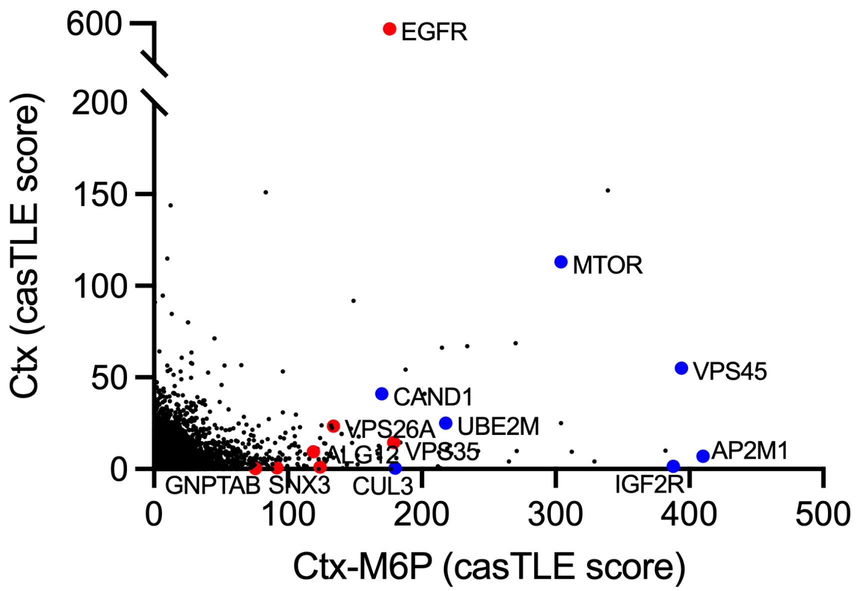


**Fig. S6**. **Comparison of hits in Ctx and Ctx-M6Pn CRISPR KO screen.** Selected gene hits for regulation of EGFR degradation by Ctx-M6Pn vs. Ctx. Hits with positive effect are in blue, and hits with negative effect are in red.

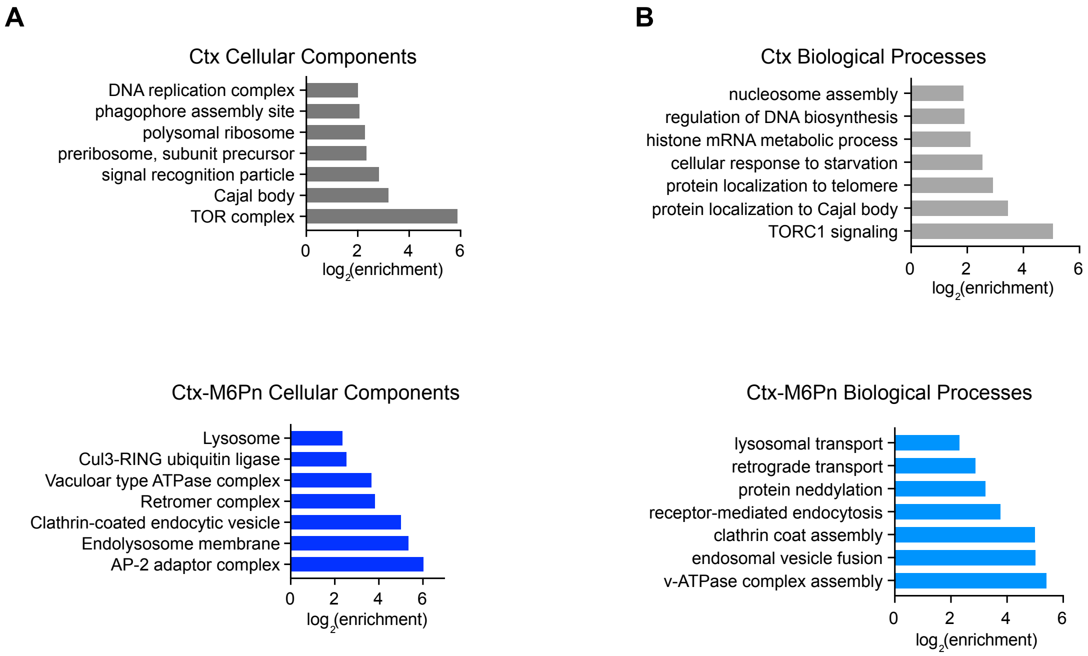


**Fig. S7**. **Gene Ontology (GO) term analysis from the genome-wide CRISPR KO screen. (A)** Gene ontology analysis of cellular components terms for Ctx and Ctx-M6Pn. **(B)** Gene ontology analysis of biological processes terms for Ctx and Ctx-M6Pn.

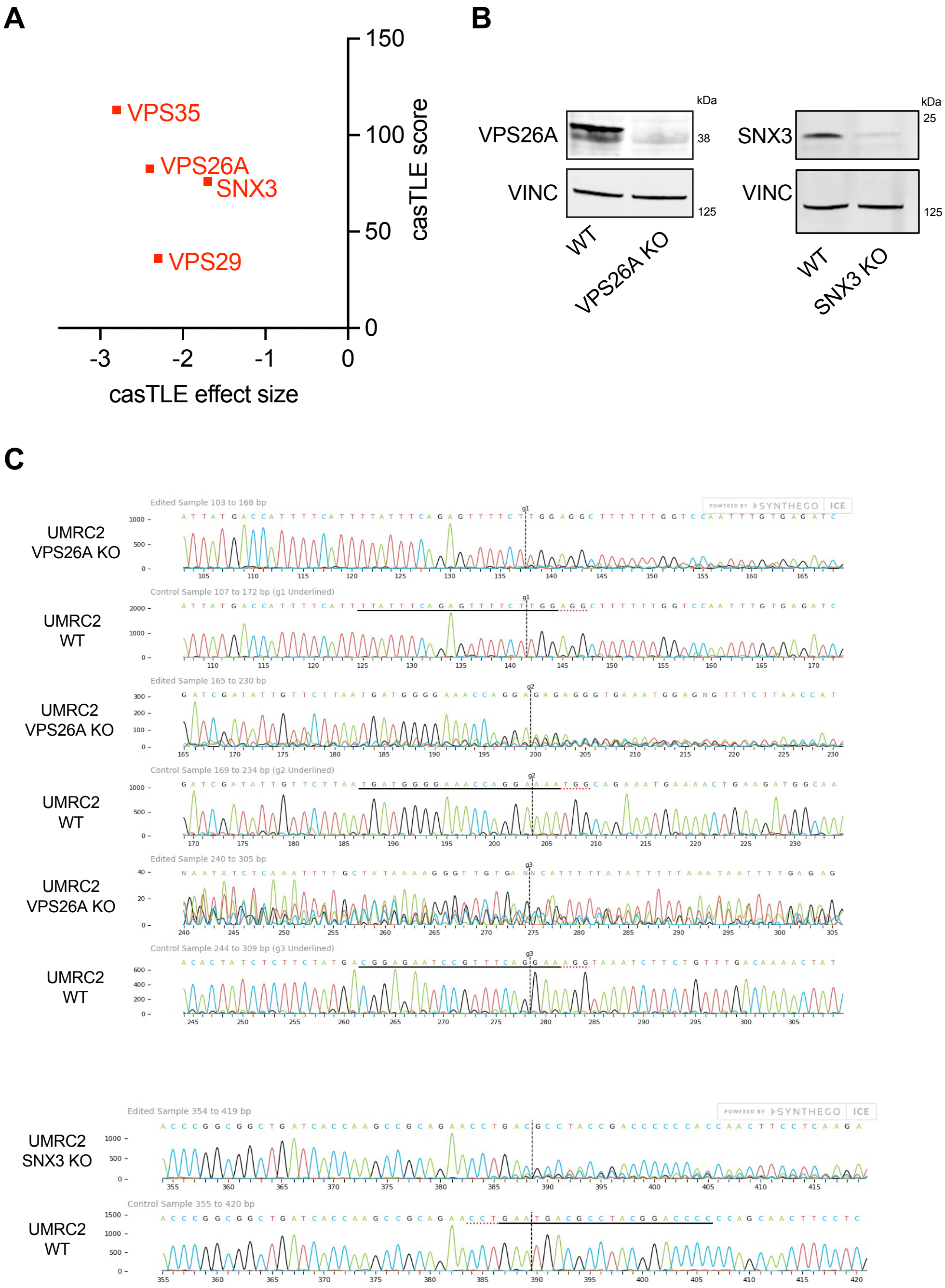


**Fig. S8**. **Retromer complex genes are negative hits from the CRISPR screen. (A)** CasTLE score and negative effect size of retromer complex genes. **(B)** Immunoblot analysis of knockout of VPS26A and SNX3. **(C)** UMRC2 knockout versus wild-type chromatograms. DNA sequences targeted by gRNAs are underlined.

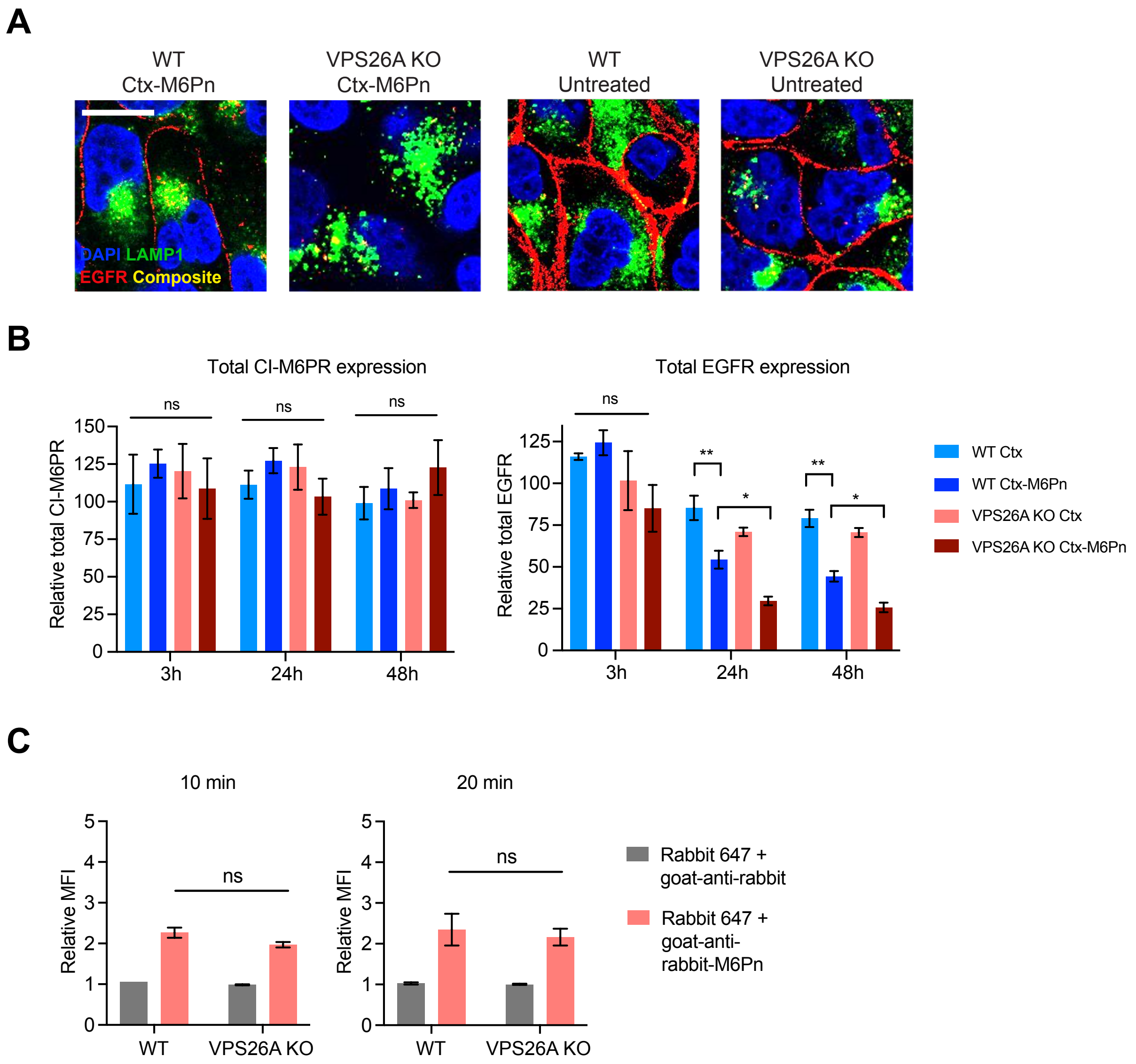


**Fig S9. Disruption of retromer complex gene VPS26A enhances LYTAC-mediated degradation of EGFR but does not affect cargo internalization or total CI-M6PR expression.** **(A)** Visualization of EGFR degradation in WT and VPS26A KO cells via confocal microscopy following continuous treatment with 10 nM Ctx-M6Pn for 48 h. Scale bar, 20 µm. **(B)** Immunoblot analysis of CI-M6PR (left panel) and EGFR (right panel) levels following 3, 24, or 48 h treatment of 10 nM Ctx or Ctx-M6Pn in WT or VPS26A KO cells. **(C)** Mean fluorescence intensity (MFI) relative to the control (rabbit IgG-647 only) for WT, VPS26A KO and SNX3 KO cells incubated at 37 °C for 10 or 20 mins with 50 nM rabbit IgG-647 and 25 nM goat anti-rabbit or goat anti-rabbit M6Pn. MFI was determined by live-cell flow cytometry. Data in **(A)** are representative of two independent experiments. For **(B)** and **(C),** data represent three independent experiments, and data are shown as mean ± S.E.M. P values were determined by unpaired two-tailed t-tests.

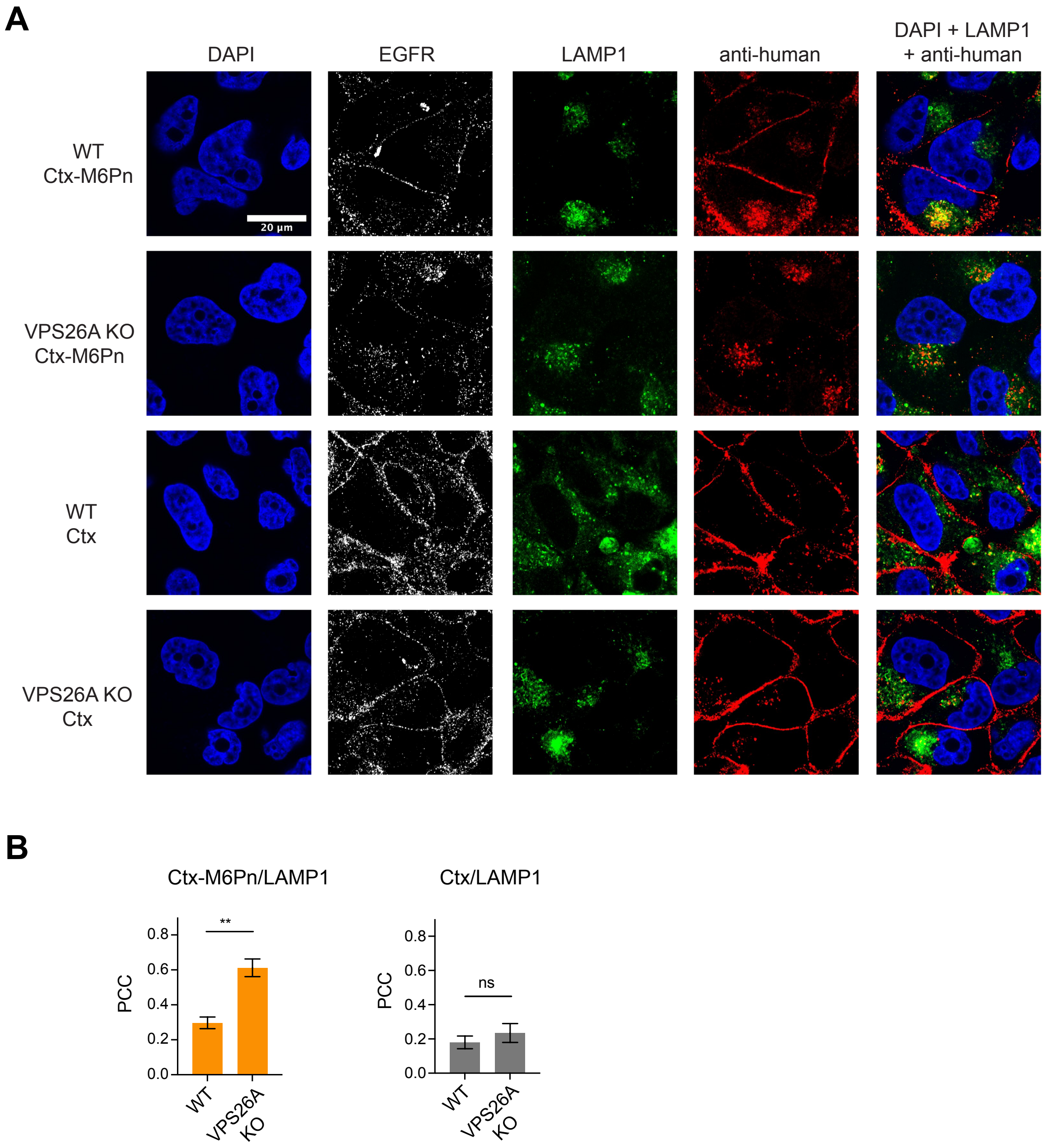


**Fig. S10. LYTACs are absent on the cell-surface in retromer knockout cells. (A)** Localization of EGFR and Ctx-M6Pn or Ctx (goat anti-human-647) in WT and VPS26A KO cells with EGFR or LAMP1 after pulse treatment of Ctx-M6Pn. Cells were treated with 10 nM Ctx-M6Pn for 24 h, then washed and incubated with fresh media for additional 24 h. Scale bar, 20 µm. **(B)** PCC analysis for LAMP1 and LYTAC colocalization in WT and VPS26A KO cells in Fig. 3F from three independent images.

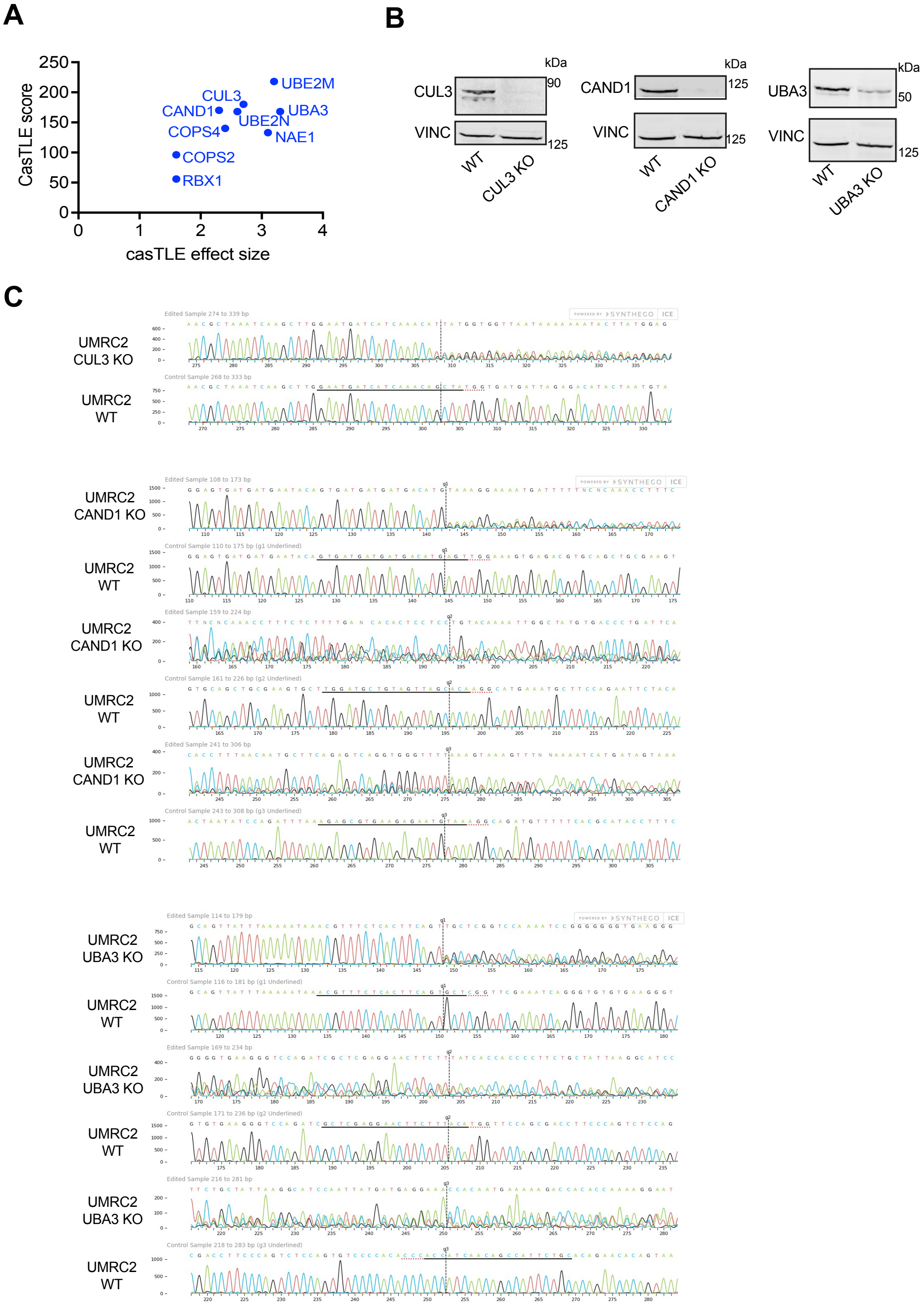


**Fig. S11**. **Genes involved in neddylation of CUL3 are positive hits from the CRISPR screen.** **(A)** CasTLE score and maximum positive effect size of CUL3-neddylation genes. **(B)** Immunoblot analysis of knockout of CUL3, UBA3, and CAND1. **(C)** UMRC2 knockout versus wild-type chromatograms. DNA sequences targeted by gRNAs are underlined.

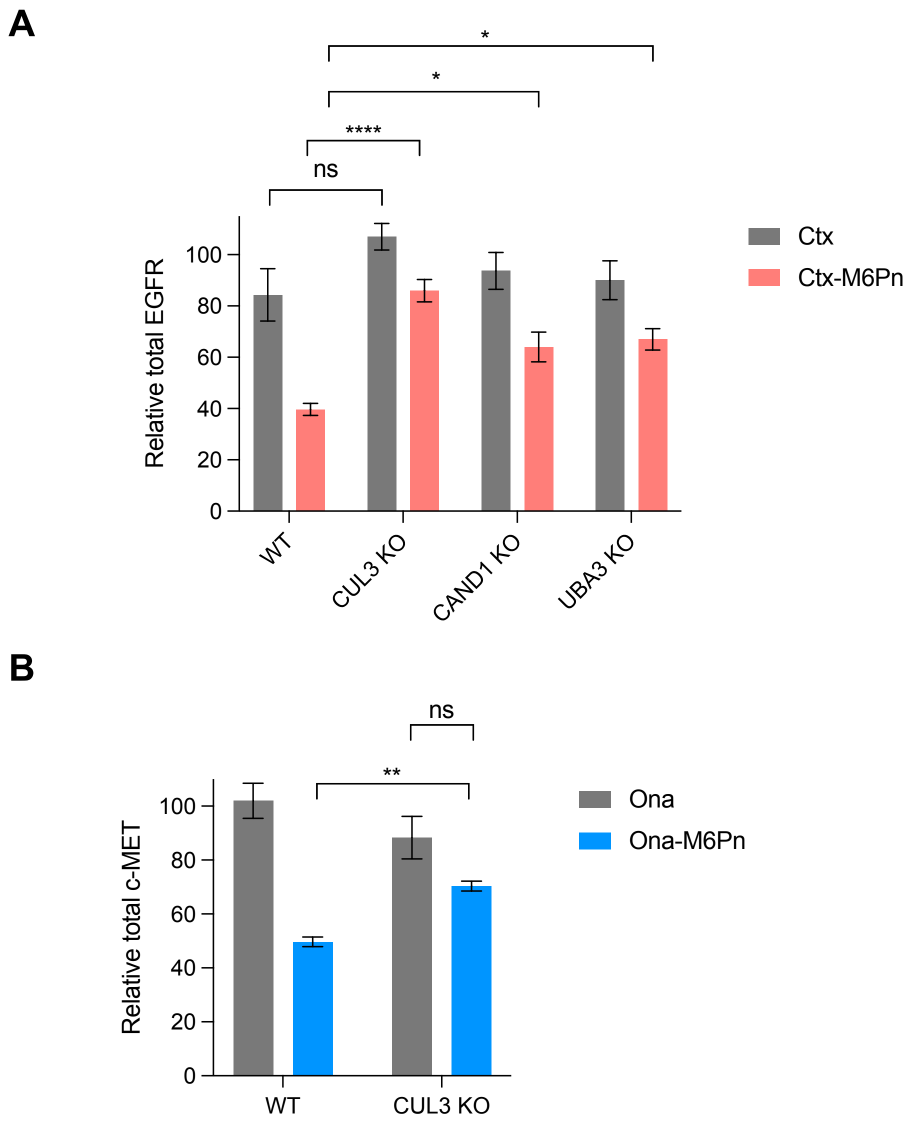


**Fig. S12**. **Knockout of CUL3-neddylation genes impair LYTAC-mediated degradation. (A)** Quantification of immunoblot of EGFR levels in WT, CAND1, and UBA3 KO cells after treatment with 10 nM Ctx or Ctx-M6Pn for 48h. **(B)** Quantification of immunoblot of c-MET levels in WT and CUL3 KO cells after treatment with Ona or Ona-M6Pn for 48h. Data represent three independent experiments, and data are shown as mean ± S.E.M. *P* values were determined by unpaired two-tailed *t*-tests. NS, not significant.

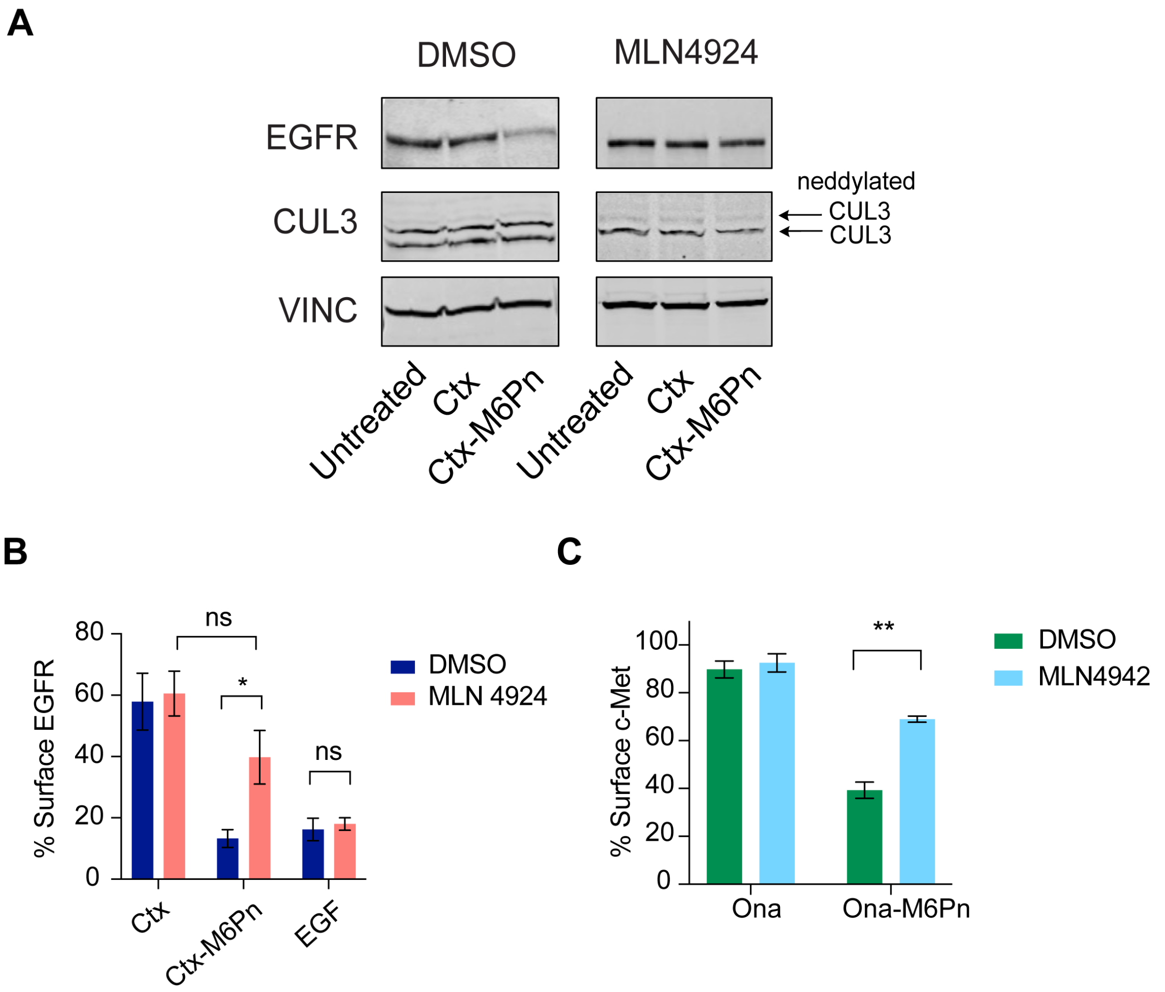


**Fig. S13. Cycling of neddylation is essential for LYTAC activity. (A)** Immunoblot analysis of EGFR levels in UMRC2 cells pre-treated with DMSO or MLN4924 (2 µM) for 24 hours, then treated with 10 nM Ctx or Ctx-M6Pn for 24 h. **(B)** Degradation of cell-surface EGFR in HeLa cells pre-treated with DMSO or MLN4924 (2 µM) for 24 hours, then treated with 10 nM Ctx or Ctx-M6Pn for 24 h or 50 nM EGF for 1 h as determined by live-cell flow cytometry. **(C)** Degradation of cell-surface c-Met in HeLa cells pre-treated with DMSO or MLN4924 (2 µM) for 24 hours, then treated with 10 nM Ona or Ona-M6Pn for 24 h as determined by live-cell flow cytometry.

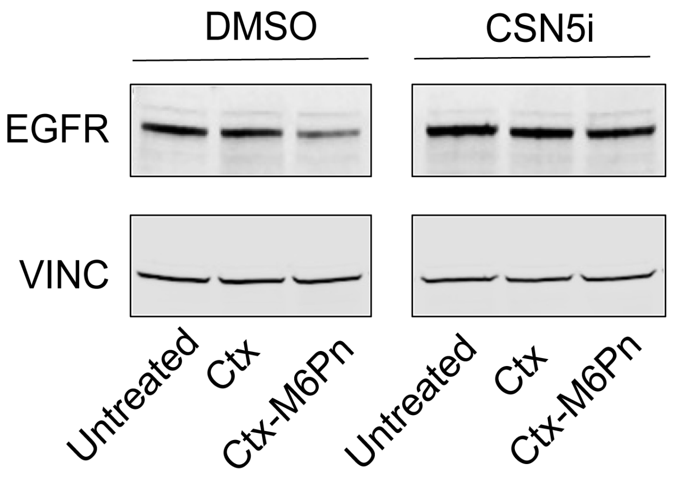


**Fig. S14. Chemical inhibition of deneddylation attenuates CI-M6PR-mediated degradation.** Immunoblot analysis of EGFR levels in UMRC2 cells pre-treated with DMSO or CSN5i (1 μM) for 24 hours, then treated with 10 nM Ctx or Ctx-M6Pn for 24 h.

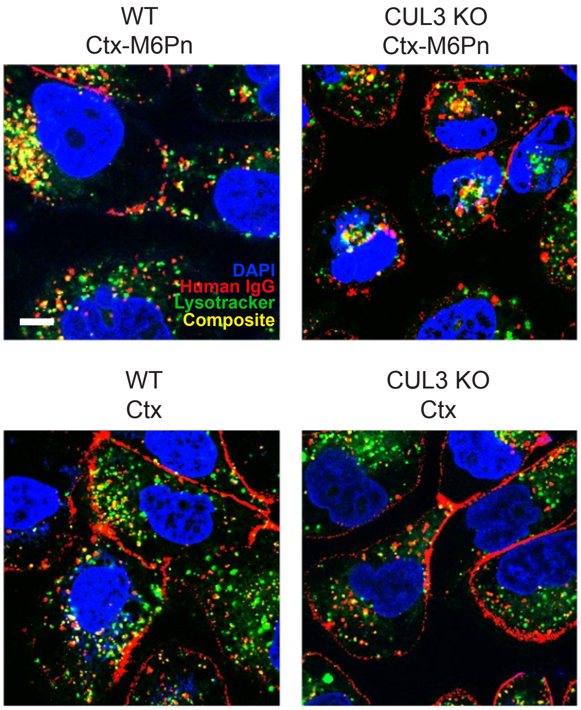


**Fig. S15. Internalization of EGFR targeting LYTAC in WT and CUL3 KO cells**. Visualization of WT or CUL3 KO cells following 1.5 h incubation at 37°C with 50 nM human IgG-647 and 25 nM Ctx or Ctx-M6Pn. Images are representative of two independent experiments. Scale bar, 10 µm.

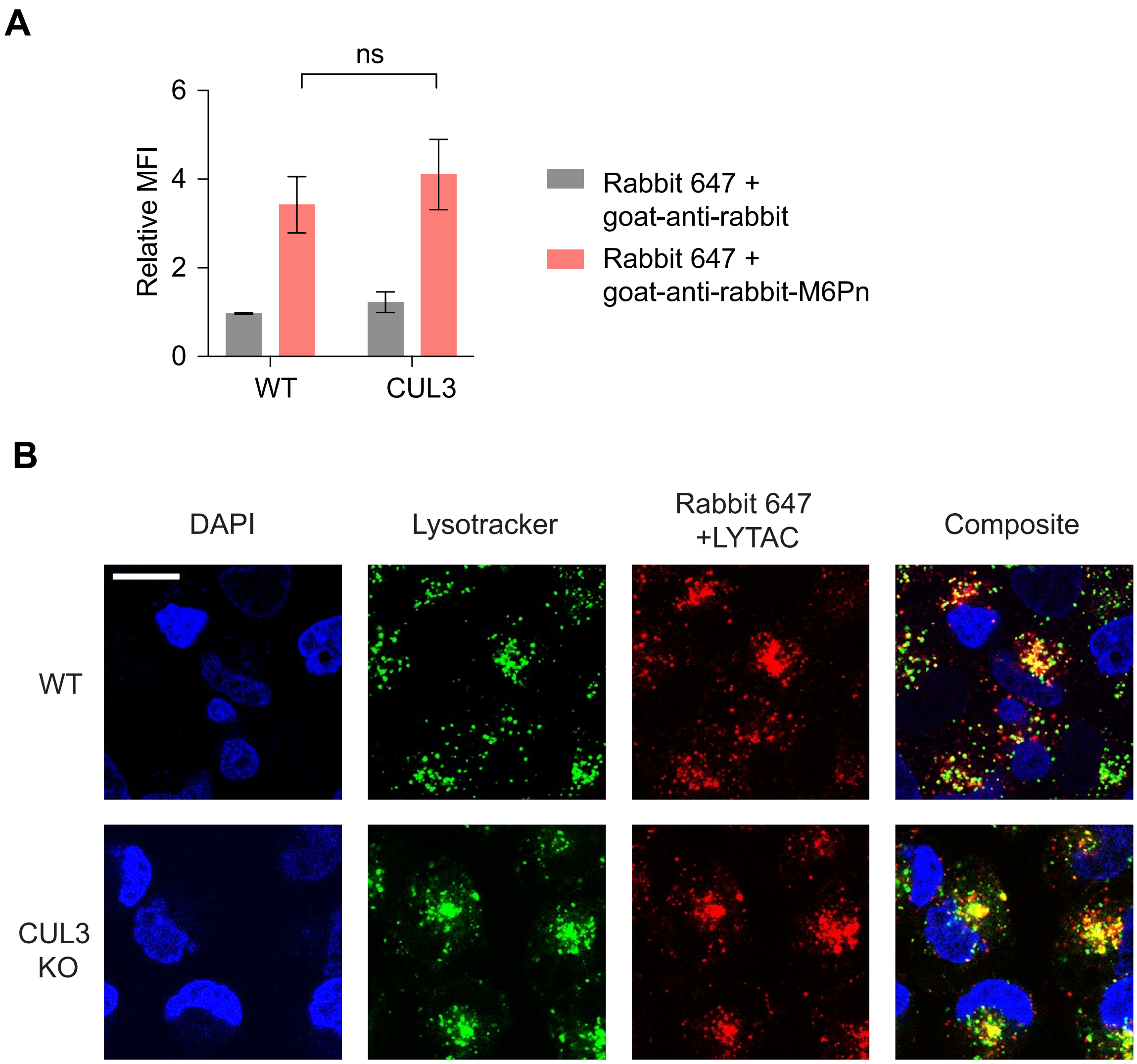


**Fig S16. Internalization efficiency of soluble cargos does not change in CUL3 KO cells compared to WT cells. (A)** Uptake of rabbit IgG-647. Mean fluorescence intensity (MFI) relative to the control (rabbit IgG-647 only) for WT and CUL3 KO cells incubated at 37 °C for 1 h with 50 nM rabbit IgG-647 and 25 nM goat anti-rabbit or goat anti-rabbit M6Pn. MFI was determined by live-cell flow cytometry. **(B)** Live-cell imaging of UMRC2 WT and CUL3 KO cells that were incubated at 37 °C for 1 h with 50 nM rabbit IgG-647 and 25 nM goat anti-rabbit M6Pn (LYTAC). Images are representative of two independent experiments. Scale bar, 20 µm.

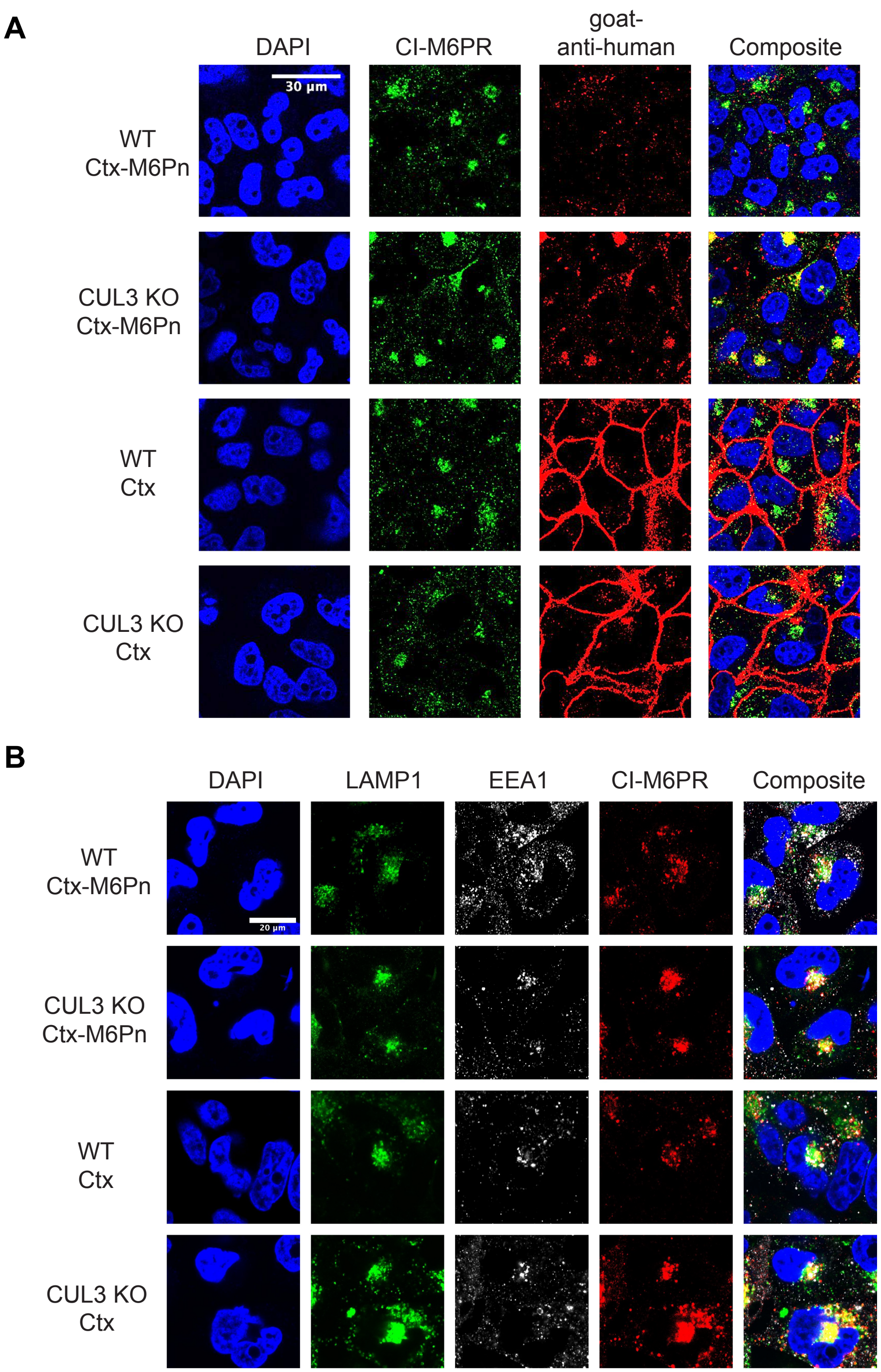


**Fig. S17. Localization of Ctx in WT and CUL3 KO cells.** **(A)** Visualization of CI-M6PR and Ctx or Ctx-M6Pn (goat anti-human-647) in WT and CUL3 KO cells after pulse treatment. Cells were treated with 10 nM Ctx or Ctx-M6Pn for 24h, washed and incubated with fresh media for additional 48 h. **(B)** Localization of CI-M6PR with LAMP1 and EEA1 in WT and CUL3 cells after pulse treatment. Cells were treated with 10 nM Ctx or Ctx-M6Pn for 24h, washed and incubated with fresh media for additional 48 h. Images are representative of two independent experiments.

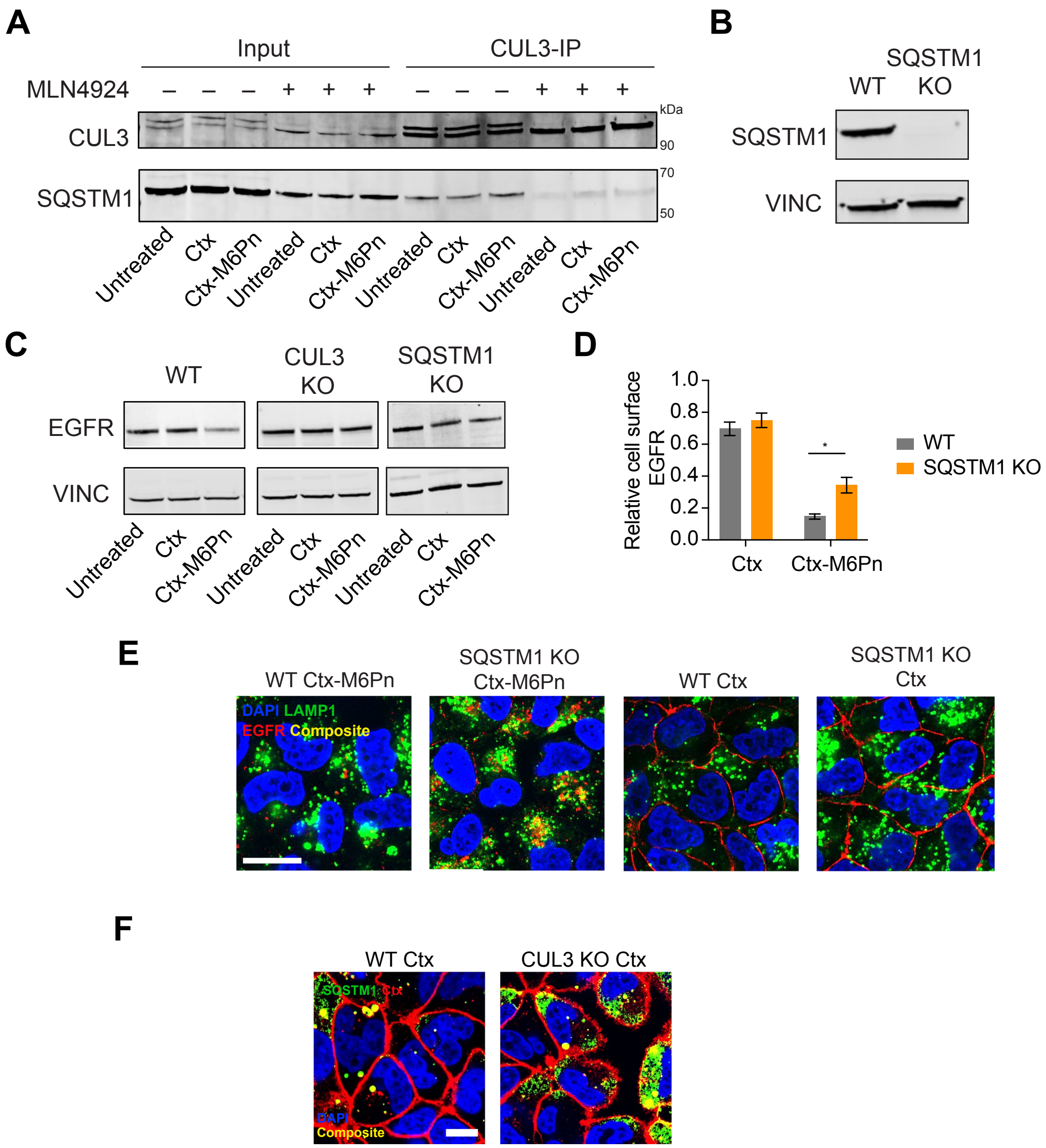


**Fig. S18. SQSTM1 is essential for late endosomal maturation of LYTAC complexes. (A)** Immunoprecipitation of CUL3 in cells pre-treated with DMSO or MLN4924 (2 μM) for 24 hours and then treated with 10 nM Ctx or Ctx-M6Pn for 24h. **(B)** Immunoblot analysis of SQSTM1 knockout in UMRC2 cells. **(C)** Immunoblot analysis of EGFR levels in WT, CUL3, and SQSTM1 KO cells after treatment with 10 nM Ctx or Ctx-M6Pn for 48h. **(D)** Degradation of cell-surface EGFR in WT and SQSTM1 KO cells as determined by live-cell flow cytometry following 48h treatment of 10 nM of Ctx or Ctx-M6Pn. **(E)** Visualization of EGFR degradation in WT and SQSTM1 KO cells via confocal microscopy following continuous treatment with Ctx or Ctx-M6Pn for 72h. Scale bar, 20 μm. **(F)** Visualization of SQSTM1 and anti-human (Ctx) in WT or CUL3 KO cells via confocal microscopy following 48h treatment with Ctx. Scale bar, 10 μm. Data in **(D)** are shown as mean ± S.E.M. *P* values were determined by unpaired two-tailed *t*-tests. Data in **(A), (B), (E), are (F)** are representative of two independent experiments.

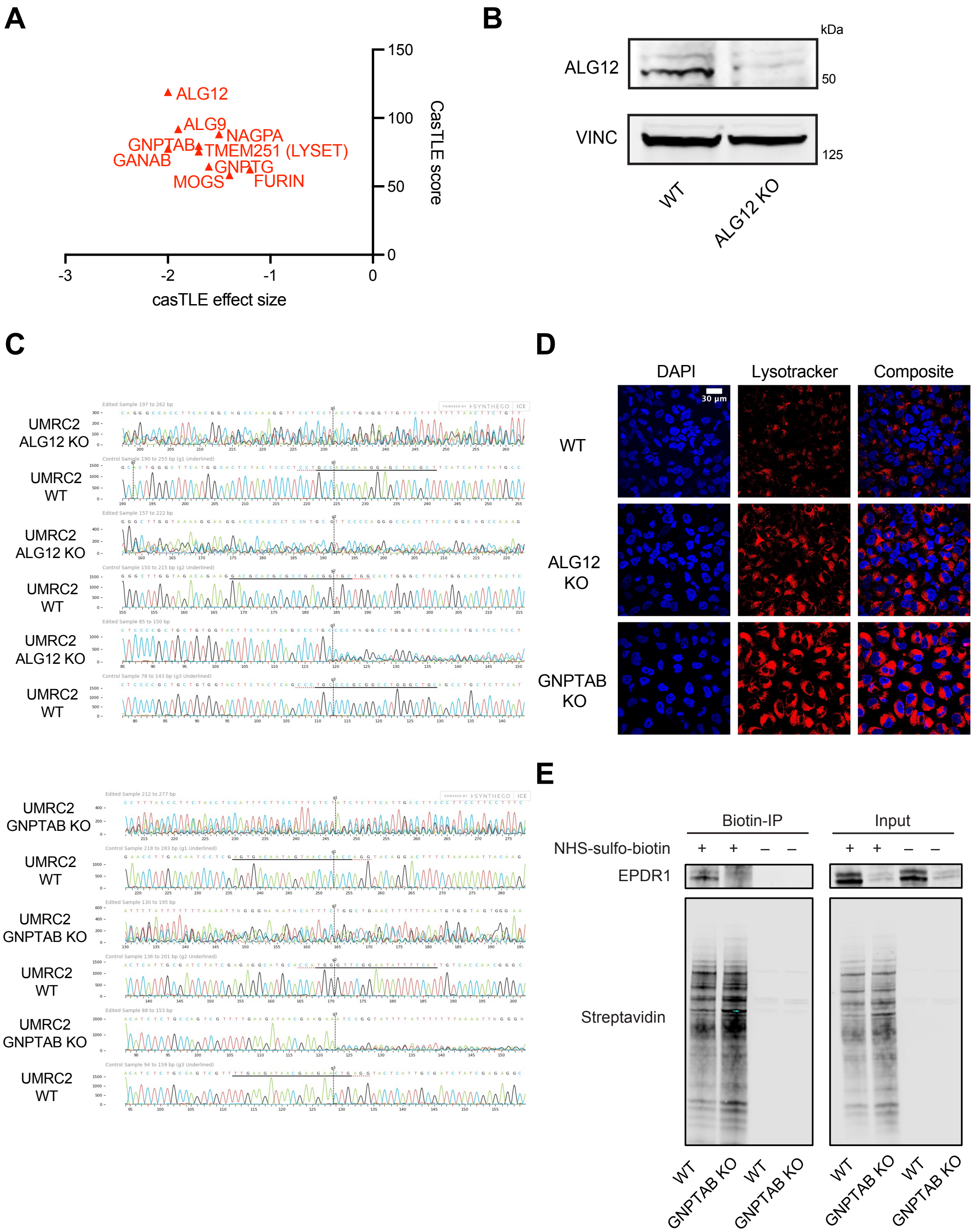


**Fig. S19. Genes involved in M6P biosynthesis pathway. (A)** CasTLE score and minimum negative effect size of genes involved in M6P biosynthesis pathway. **(B)** Immunoblot analysis of ALG12 knockouts in UMRC2 cells. **(C)** UMRC2 knockout versus wild-type chromatograms. DNA sequences targeted by gRNAs are underlined. **(D)** Lysotracker staining in WT, ALG12 KO, and GNPTAB KO cells by confocal microscopy. **(E)** Immunoblot of EPDR1 and streptavidin following biotin enrichment. WT or GNPTAB KO cells were incubated in PBS (no biotin control) or 1 mg/ml solution of NHS-sulfo-biotin in PBS with gentle rocking for 30 minutes at 4°C. Lysates were harvested and enriched with streptavidin beads.

| **Gene** | **Indel %** | **Knockout-score** |
|---|---|---|
| CUL3 | 77 | 56 |
| UBA3 | 100 | 100 |
| CAND1 | 100 | 99 |
| SNX3 | 97 | 92 |
| VPS26A | 94 | 91 |
| ALG12 | 98 | 89 |
| GNPTAB | 98 | 98 |
| SQSTM1 | 99 | 99 |

**Supplementary Table 1. Knockout sequencing analysis**

| Channel | Sample | | Channel | Sample |
|---|---|---|---|---|
| 126 | WT Ctx 1 | | 127N | KO Ctx 1 |
| 127C | WT Ctx 3 | | 128N | KO Ctx 3 |
| 128C | WT NT 1 | | 129N | KO NT 1 |
| 129C | WT NT 2 | | 130N | KO NT 2 |
| 130C | WT NT 3 | | 131N | KO NT 3 |
| 131C | WT M6P 1 | | 132N | KO M6P 1 |
| 132C | WT M6P 2 | | 133N | KO M6P 2 |
| 133C | WT M6P 3 | | 134N | KO M6P 3 |

**Supplementary Table 2: TMT Labeling Conditions**

| Antibody | Source (#) | Usage, Dilution |
|---|---|---|
| Rabbit IgG | Bio X Cell (BE0095) | Functional |
| Goat-Anti-Rabbit IgG | Jackson ImmunoResearch (111-005-144) | Functional |
| Rabbit-anti-EGFR | Cell Signaling Technology (D38B1) | WB 1:1000, IF 1:100 |
| Mouse anti-Vinculin | Bio-Rad (V284) | WB, 1:1000 |
| Mouse-anti-EGFR | Invitrogen (MA513319) | Flow Cytometry, 10 μg/ml |
| Cetuximab | Eli Lilly | Functional |
| Onartuzumab | ProSci (10-454) | Functional |
| Mouse-anti-CA9 | R&D Systems (MAB2188) | Flow Cytometry, 10 μg/ml |
| Rabbit anti-CA9 | Proteintech (11071-1-AP) | WB 1:1000 |
| Rabbit-anti-cMET | Cell Signaling Technology (D1C2) | WB 1:1000 |
| Goat-anti-CMET | Biotechne (AF276) | Flow Cytometry, 10 μg/ml |
| Anti-CI-M6PR 488 | Abcam (ab205812) | Flow Cytometry, 10 μg/ml, IF 1:100 |
| Anti-CI-M6PR 647 | Abcam (ab205813) | Flow Cytometry, 10 μg/ml, IF 1:100 |
| Mouse anti-LAMP1-488 | Thermo Scientific (MA5-18121) | IF 1:100 |
| Mouse anti-LAMP2-488 | eBioscience H4B4 | IF 0.25 μg/ml |
| Rabbit-anti-CUL3 | Cell Signaling Technology (2759) | WB 1:1000 |
| Rabbit-anti-UBA3 | Novus (NBP2-49443) | WB 0.4 μg/ml |
| Rabbit-anti-CAND1 | Cell Signaling Technology (D1F2) | WB 1:1000 |
| Rabbit-anti-CUL3 | Novus (NB100-58788) | IP 4 μg/ml |
| Mouse-anti-VPS26A | Novus (NBP2-36754) | WB 1 μg/ml |
| Rabbit-anti-SNX3 | Proteintech (10772-1-AP) | WB 1:500 |
| Rabbit-anti-ALG12 | Novus (NBP2-86977) | WB 1 μg/ml |
| Rabbit-anti-GNPTAB | Bioss (BS-13476R) | WB 1:500 |
| Mouse-anti-EPDR1 | Novus (H00054749-M01) | Flow Cytometry, 10 μg/ml |
| Rabbit-anti-GNS | Proteintech (13044-1-AP) | Flow Cytometry, 10 μg/ml |
| Rabbit-anti-BGAL | Proteintech (15518-1-AP) | Flow Cytometry, 10 μg/ml |
| IRDye 800CW Goat-anti-rabbit IgG (H+L) | LI-COR (926-32211) | WB, 1:10000 |
| IRDye 800CW Goat-anti-mouse IgG (H+L) | LI-COR (926-32210) | WB, 1:10000 |
| Goat anti-human IgG-Alexa Fluor 647 | Jackson ImmunoResearch (109-605-003) | Flow Cytometry, IF, 1:375 |
| Goat anti-mouse IgG-Alexa Fluor 647 | Jackson ImmunoResearch (115-605-071) | Flow cytometry, IF, 1:375 |
| Goat anti-rabbit IgG (H+L) Alexa Fluor 568 | Invitrogen (A11011) | IF 1:250 |

**Supplementary Table 3: Antibody information and concentrations**

**Supplementary Note: Chemical Synthesis Procedures and Characterization**

Chemical Synthesis Procedures

Synthesis of M6Pn-3

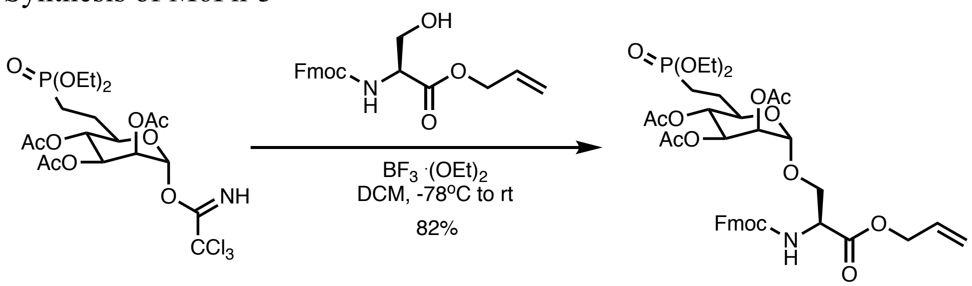


**M6Pn-1** **M6Pn-2**

**M6Pn-2**: To a flame dried flask was added Fmoc-Allyl-Ser(OH) (1.18 g, 3.21 mmol, 1.21 eq), **M6Pn-1**[7] (1.55 g, 2.66 mmol, 1.0 eq), and freshly activated 4Å molecular sieves (2.00 g, dried overnight, then flame dried for 2-4 hours), and $CH_2Cl_2$ (40 ml, 0.067M). The reaction was cooled to -78°C for 20 minutes then boron trifluoride diethyletherate (0.33 ml, 2.66 mmol, 1.0 eq) was added dropwise. The reaction was allowed to warm up to room temperature gradually over 16 hours. Upon completion, the reaction was quenched with triethylamine (1ml), filtered over celite and concentrated. The resulting residue was purified by flash column chromatography on silica gel (40→100% ethyl acetate in hexane) to give a white foam (82% yield). $^{1}$H NMR (400 MHz, Methanol-$d_4$) δ 7.81 (d, $J$ = 7.5 Hz, 2H), 7.70 (d, $J$ = 7.6 Hz, 2H), 7.40 (t, $J$ = 7.4 Hz, 2H), 7.36 – 7.25 (m, 2H), 5.97 (ddt, $J$ = 16.2, 10.9, 5.7 Hz, 1H), 5.41 – 5.31 (m, 1H), 5.29 – 5.18 (m, 3H), 5.05 (t, $J$ = 9.8 Hz, 1Hff, $J$ = 15.9, 14.5, 7.1 Hz, 3H), 4.57 (t, $J$ = 5.1 Hz, 1H), 4.44 – 4.30 (m, 2H), 4.25 (t, $J$ = 6.8 Hz, 1H), 4.04 (dtd, $J$ = 10.7, 7.4, 6.7, 3.3 Hz, 5H), 3.89 (dd, $J$ = 9.9, 6.4 Hz, 2H), 2.12 (s, 3H), 2.02 (s, 3H), 1.96 (s, 3H), 1.89 – 1.74 (m, 2H), 1.74 – 1.58 (m, 2H), 1.36 – 1.20 (t, 6H). $^{13}$C NMR (126 MHz, $CD_3OD$) δ 171.64, 171.24, 158.39, 145.21, 145.11, 142.49, 133.19, 128.80, 128.20, 126.32, 125.04, 122.98, 120.93, 120.45, 118.55, 98.89, 78.39, 72.76, 72.63, 71.76, 69.75, 68.21, 66.95, 63.42, 63.31, 63.26, 55.60, 49.51, 49.34, 49.17, 49.00, 48.83, 48.66, 48.49, 48.24, 29.51, 25.32, 25.21, 21.53, 20.65, 20.39, 16.73, 16.68. $^{31}$P NMR (162 MHz, Methanol-$d_4$) δ 32.64. ESI-HRMS Calc'd [M+H$^{+}$]=790.2834; found 790.2826.

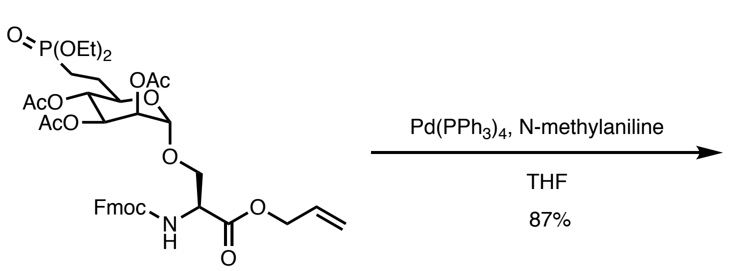


**M6Pn-2** **M6Pn-3**

**M6Pn-3**: To M6Pn-2 (0.85 g, 1.08 mmol, 1 eq) was added THF (10 ml, 0.1 M), tetrakis(triphenylphosphine)palladium (0.12 g, 0.11 mmol, 0.10 eq) , and N-methylaniline (1.17 ml, 10.8 mmol, 10 eq). The reaction was allowed to stir at room temperature for 4 hours. Upon completion, the reaction mixture was concentrated under reduced pressure and purified by flash column chromatography on silica gel (40→100% EA in hexane first, followed by 0→20% MeOH in EA – product elutes at 100% EA and then again at 15% MeOH as a salt) to give a yellow foam (87% yield). $^{1}$H NMR (500 MHz, Methanol-$d_4$) δ 7.80 (d, $J$ = 7.5 Hz, 2H), 7.70 (d, $J$ = 7.5 Hz, 2H), 7.39 (t, $J$ = 7.5 Hz, 2H), 7.32 (td, $J$ = 7.4, 1.2 Hz, 2H), 5.31 – 5.20 (m, 2H), 5.05 (t, $J$ = 9.9 Hz, 1H), 4.50 – 4.37 (m, 2H), 4.31 – 4.20 (m, 2H), 4.10 – 3.99 (m, 6H), 3.89 (q, $J$ = 7.9, 6.0 Hz, 2H), 2.11 (s, 3H), 1.98 (d, $J$ = 8.6 Hz, 3H), 1.94 (s, 3H), 1.87 – 1.73 (m, 3H), 1.70 – 1.54 (m, 1H), 1.30 – 1.21 (m, 6H). $^{13}$C NMR (101 MHz, Methanol-$d_4$) δ 170.20, 170.08, 143.97, 143.76, 141.16, 127.44, 126.86, 125.04, 124.91, 119.58, 97.88, 69.24, 68.56, 67.73, 66.81, 61.92, 61.85, 48.27, 48.06, 47.85, 47.64, 47.42, 47.21, 47.00, 46.96, 23.86, 19.25, 19.23, 15.36, 15.30, 13.10. $^{31}$P NMR (162 MHz, Methanol-$d_4$) δ 32.70. ESI-HRMS Calc'd [M-H$^{+}$]=748.2376; found 748.2370.

Solid phase peptide synthesis of M6Pn peptide

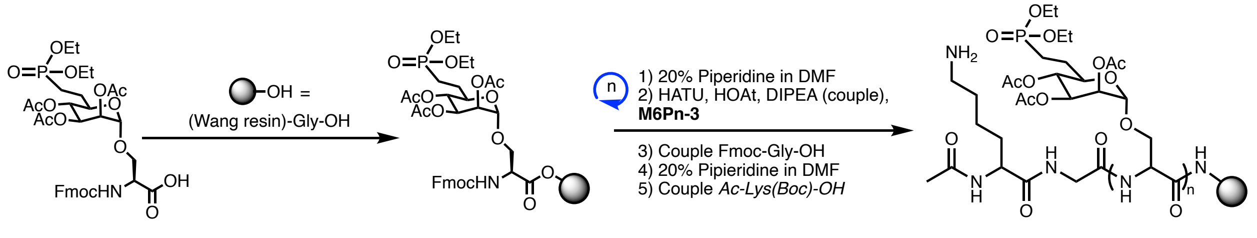


**M6Pn-3**

Solid phase peptide synthesis was performed on the CSBio CS336X peptide synthesizer. Standard Fmoc conditions were used to generate M6Pn peptides. Briefly, Fmoc-Gly-Wang-resin (1 eq) was loaded onto the synthesizer. HATU (5 eq), HOAt (hydroxy-7-azabenzotriazole) (5 eq), and DIPEA (15 eq) were used to couple M6Pn-3 (5 eq), Fmoc-Gly-OH (5 eq), or Ac-Lys(Boc)-OH (5 eq). Fmoc deprotections were conducted with 20% piperidine in DMF.

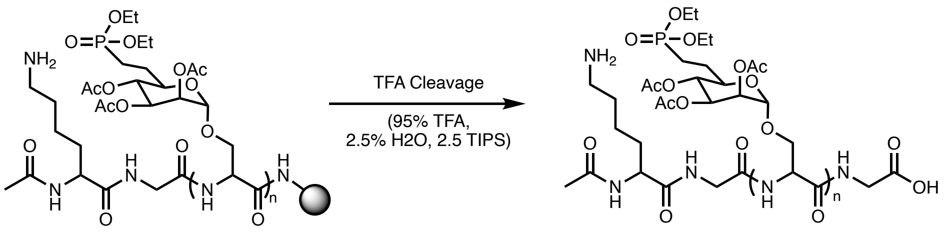


Peptides were cleaved from the resin using 2 ml of cleavage cocktail (95% TFA, 2.5% TIPS, 2.5% water). The cleaved product was concentrated under reduced pressure and deprotected (as shown below) without further purification.

Deprotection of M6Pn$_2$ and M6Pn$_5$ peptide following TFA cleavage

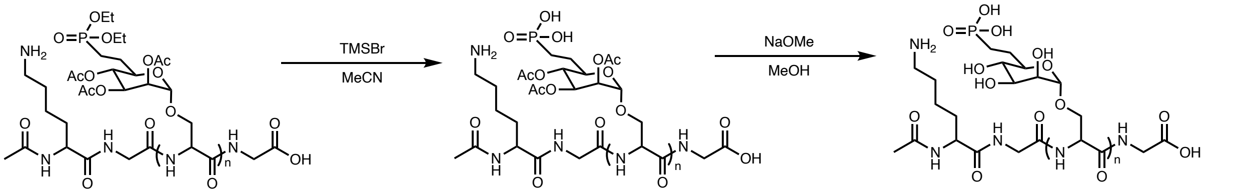


**M6Pn$_2$:** n=2, Ac-Lys-Gly-[Ser(M6Pn)]$_2$-Gly-OH
**M6Pn$_5$:** n=5, Ac-Lys-Gly-[Ser(M6Pn)]$_5$-Gly-OH

The cleaved peptide was dissolved in MeCN (3 ml, 0.03M) under $N_2$. Then TMSBr (0.3 ml, 2.25 mmol, 5 eq *per* phosphonate; 25 eq total for 5 M6Pn) was added dropwise. The $N_2$ needle was removed *immediately* and the septum was sealed with parafilm (TMSBr corrodes the needle into the reaction mixture if needle is not removed). The reaction was allowed to stir for 48 hours at room temperature and the reaction mixture was concentrated under high vacuum then freeze-dried with benzene. The dried product was dissolved in MeOH (5 ml, 8 mM) and 0.5 M sodium methoxide in methanol was added dropwise until the pH reached 9-10. The reaction was allowed to stir under $N_2$ for 24 hours. Upon completion, the reaction was quenched with formic acid, concentrated in vacuo, and purified by C18 column (elutes between 100%-90% $H_2O$ in MeCN + 0.1% TFA) to produce a white foam (82% yield from resin).

**M6Pn$_2$** ESI-HRMS Calc'd [M-2H$^{2+}$]=477.1442; found 477.1445.
Calc'd [M-H$^{+}$]=955.2975; found 955.2956
**M6Pn$_5$** ESI-HRMS Calc'd [M-2H$^{2+}$] =967.7520; found 967.7518.

BCN functionalization of M6Pn peptide

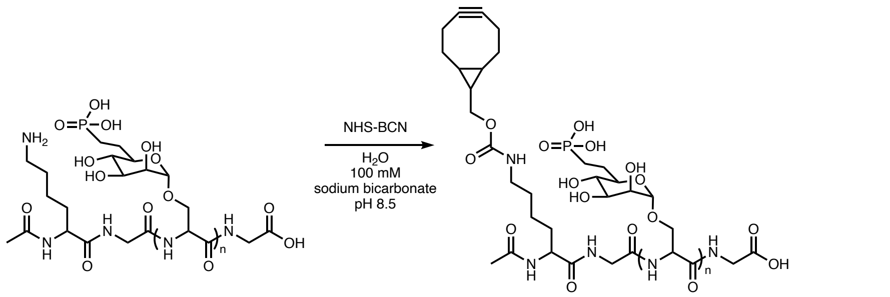


**M6Pn$_2$:** n=2, Ac-Lys-Gly-[Ser(M6Pn)]$_2$-Gly-OH
**M6Pn$_5$:** n=5, Ac-Lys-Gly-[Ser(M6Pn)]$_5$-Gly-OH

**M6Pn$_2$-BCN:** n=2, Ac-Lys(BCN)-Gly-[Ser(M6Pn)]$_2$-Gly-OH
**M6Pn$_5$-BCN:** n=5, Ac-Lys(BCN)-Gly-[Ser(M6Pn)]$_5$-Gly-OH

**M6Pn$_2$** or **M6Pn$_5$** peptide (1 eq) was dissolved in aqueous sodium bicarbonate (pH 8.5, 8 mM). BCN-NHS (6 eq) dissolved in DMSO was added to the peptide solution. DMSO was added (to ~30 mM) until the reaction mixture became clear, and the reaction was allowed to stir for 24 hours. Upon completion, the product was lyophilized and purified by C18 column.

**M6Pn$_2$-BCN** ESI-HRMS Calc'd [M-2H$^{2+}$]=565.18860; found 565.1863.
Calc'd [M-H$^{+}$]=1131.3793; found 1131.3800
**M6Pn$_5$-BCN** ESI-HRMS Calc'd [M-2H$^{2+}$] =1055.7939; found 1055.7948.

List of chemical structures

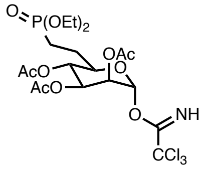

**M6Pn-1**

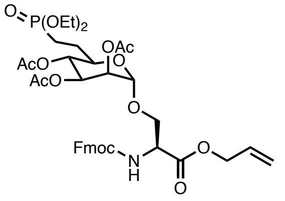

**M6Pn-2**

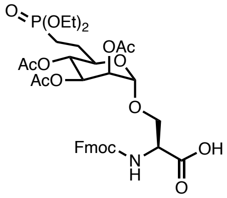

**M6Pn-3**

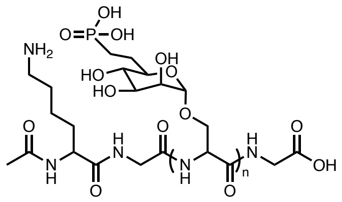

**M6Pn$_2$:** n=2, Ac-Lys-Gly-[Ser(M6Pn)]$_2$-Gly-OH
**M6Pn$_5$:** n=5, Ac-Lys-Gly-[Ser(M6Pn)]$_5$-Gly-OH

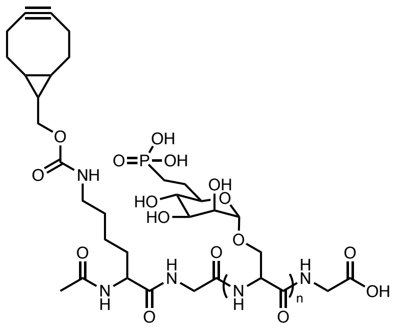

**M6Pn$_2$-BCN:** n=2, Ac-Lys(BCN)-Gly-[Ser(M6Pn)]$_2$-Gly-OH
**M6Pn$_5$-BCN:** n=5, Ac-Lys(BCN)-Gly-[Ser(M6Pn)]$_5$-Gly-OH

Characterization of peptides

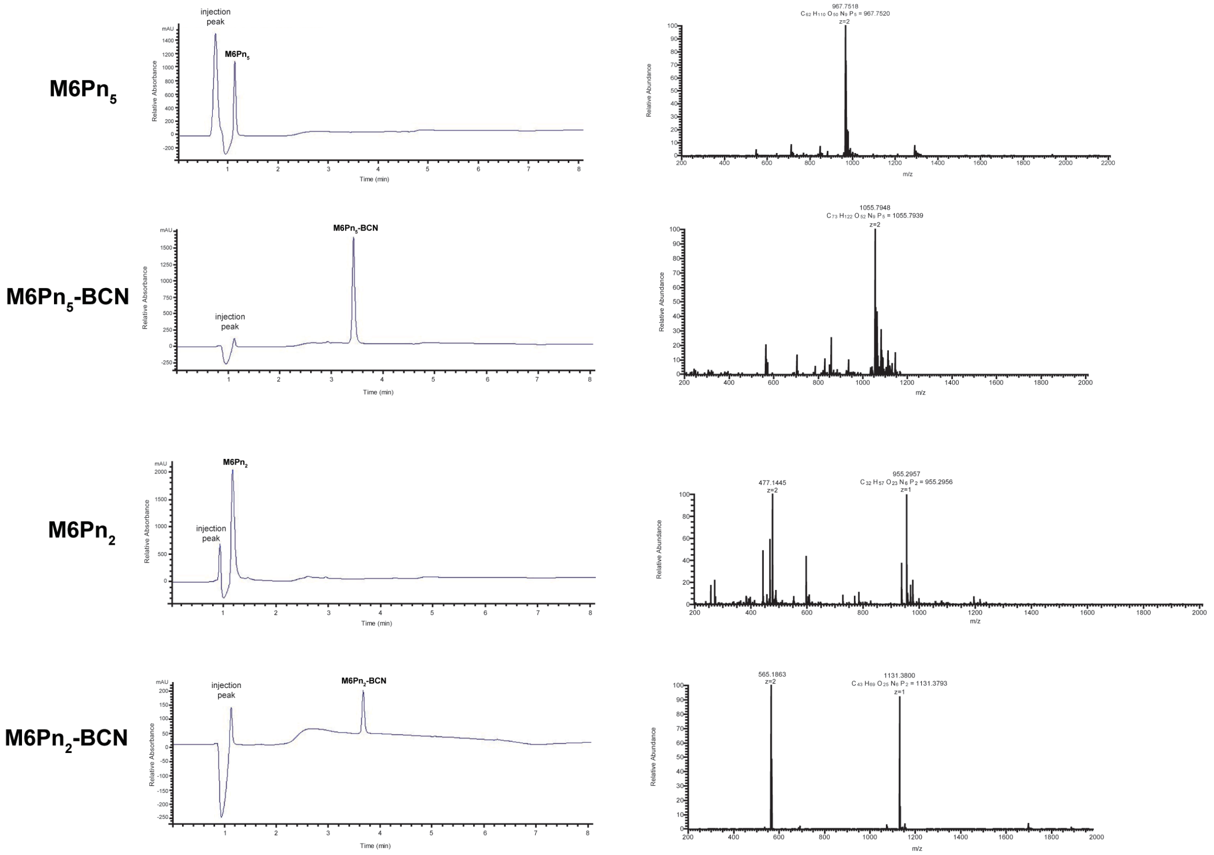


LC-MS chromatogram showing relative UV absorbance at 215 nm and mass spectrum of peptides. LC-MS method: linear gradient from 0% solvent B to 100% B over 5 minutes (solvent A: water + 0.1% TFA; solvent B; acetonitrile + 0.1% TFA).

<u>NMR Spectra</u>

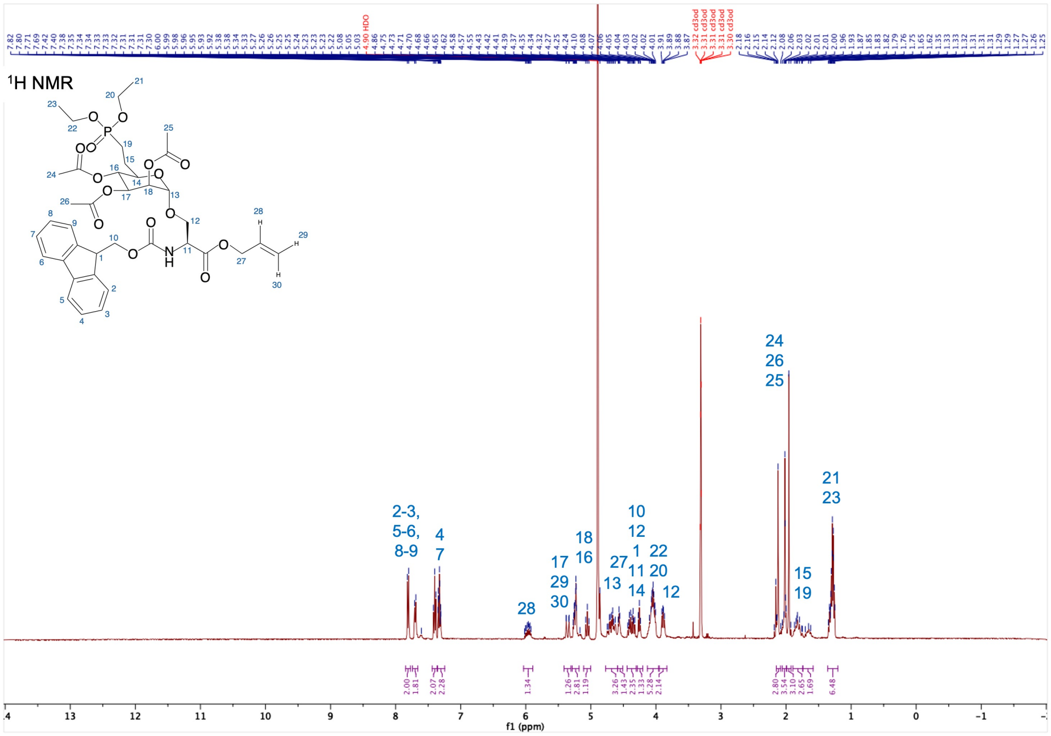


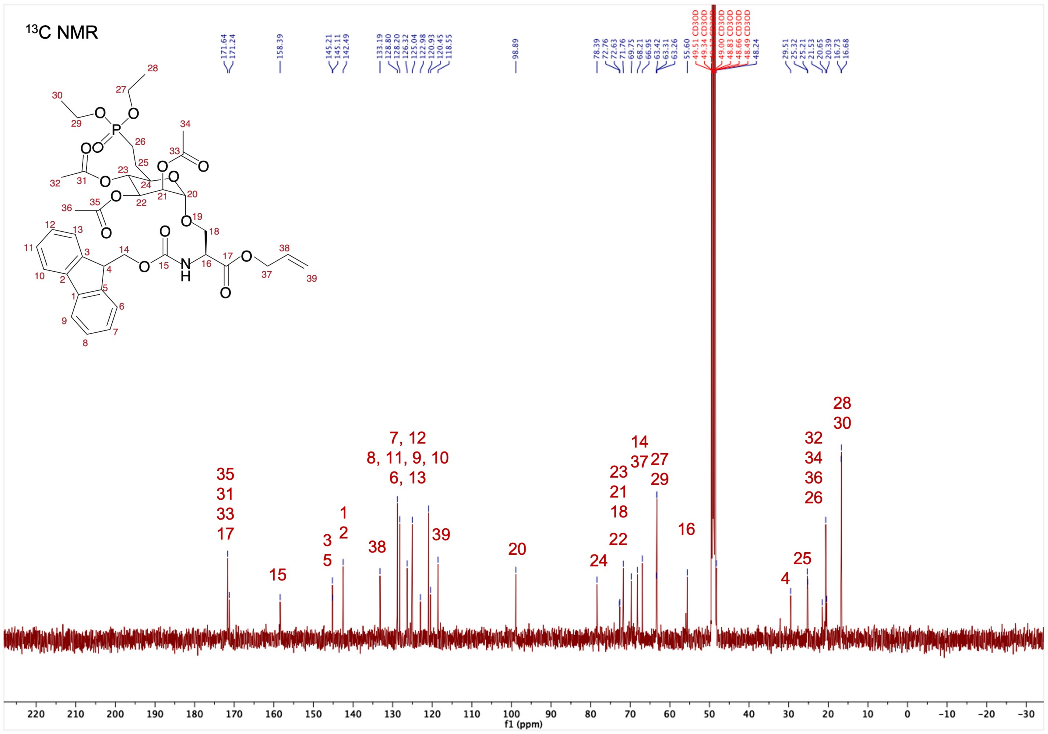

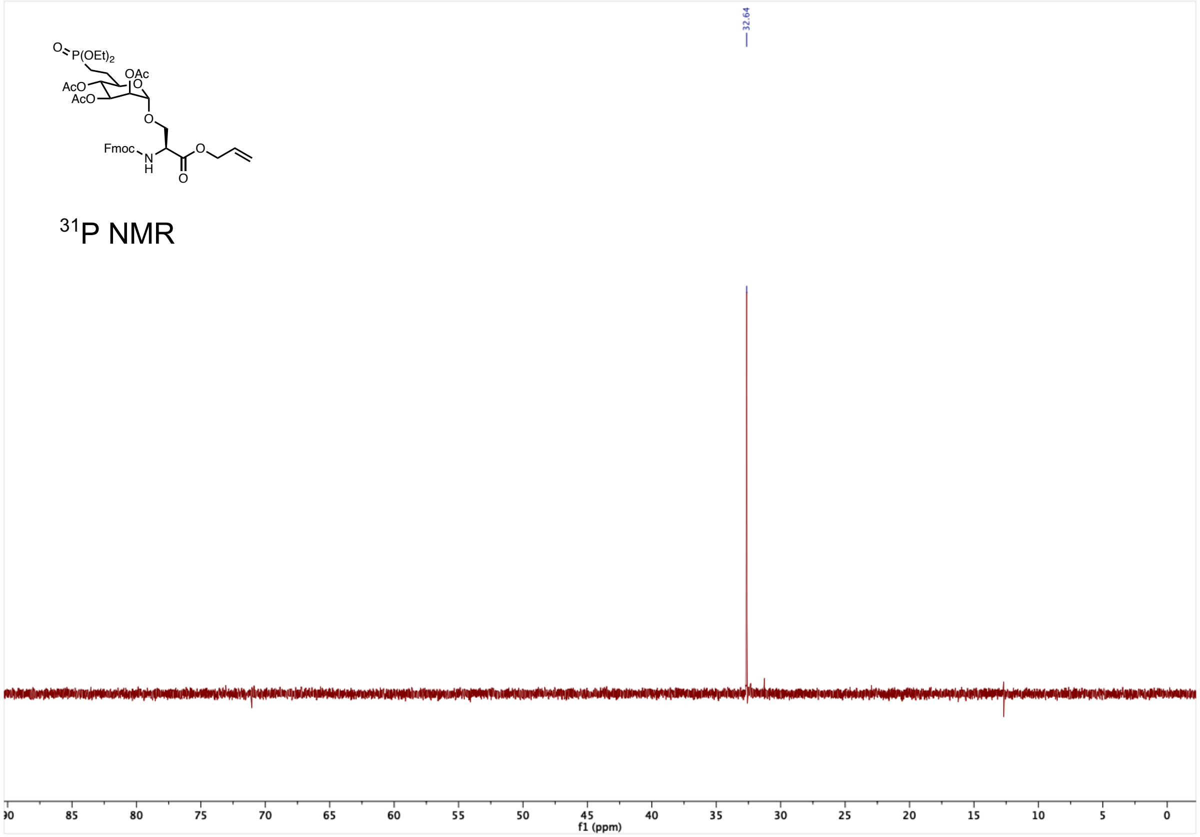

O=P(OEt)2
OAc
AcO
AcO
O
O
Fmoc
N
H
O
O
31P NMR
32.64
90 85 80 75 70 65 60 55 50 45 40 35 30 25 20 15 10 5 0
f1 (ppm)

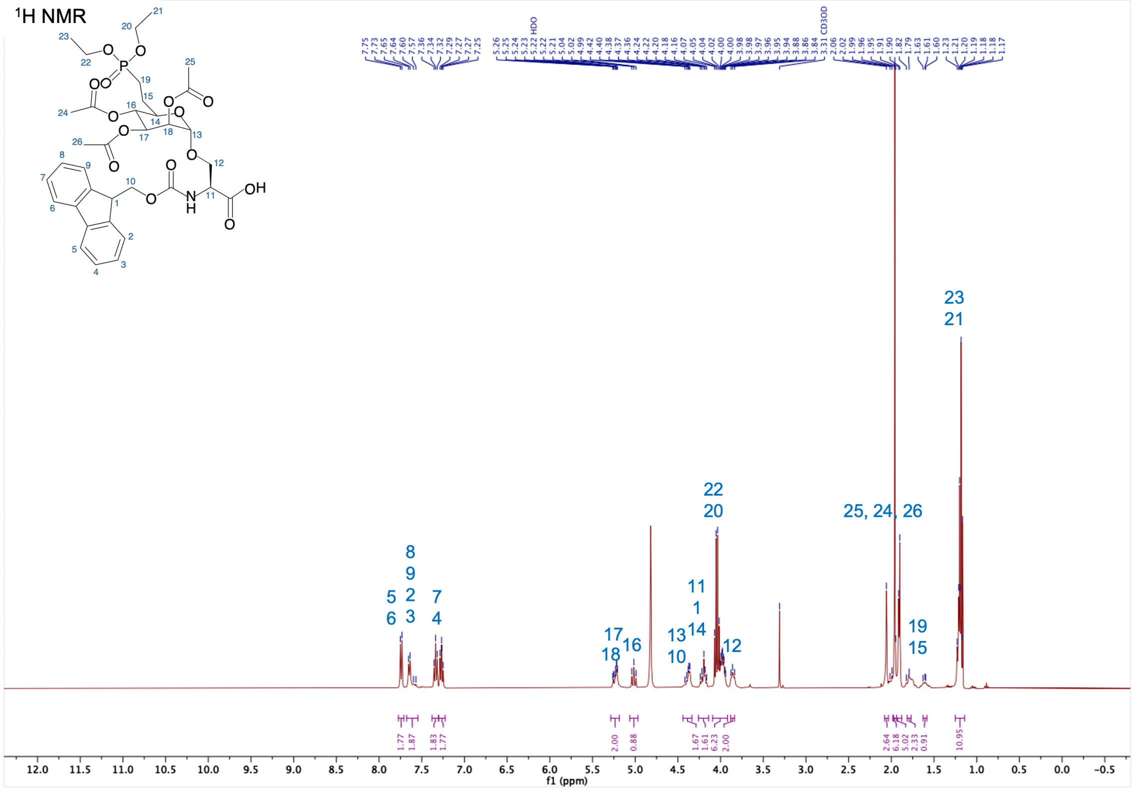

1H NMR
7.75 7.73 7.65 7.64 7.60 7.57 7.36 7.34 7.32 7.29 7.27 7.27 7.25
5.26 5.25 5.24 5.23 HDO 5.22 5.21 5.04 5.02 4.99 4.42 4.40 4.38 4.37 4.36 4.24 4.22 4.20 4.18 4.16 4.07 4.05 4.04 4.02 4.00 4.00 3.98 3.98 3.97 3.96 3.94 3.88 3.86 3.84
3.31 CD3OD
2.06 2.02 1.99 1.96 1.95 1.91 1.90 1.82 1.79 1.63 1.61 1.60 1.23 1.21 1.20 1.19 1.18 1.18 1.17
5
6
8
9
2
3
7
4
17
18
16
13
10
11
1
14
12
22
20
25, 24, 26
19
15
23
21
1.77 1.87 1.83 1.77 2.00 0.88 1.67 1.61 6.23 2.00 2.64 6.18 5.02 2.33 0.91 10.95
12.0 11.5 11.0 10.5 10.0 9.5 9.0 8.5 8.0 7.5 7.0 6.5 6.0 5.5 5.0 4.5 4.0 3.5 3.0 2.5 2.0 1.5 1.0 0.5 0.0 −0.5
f1 (ppm)


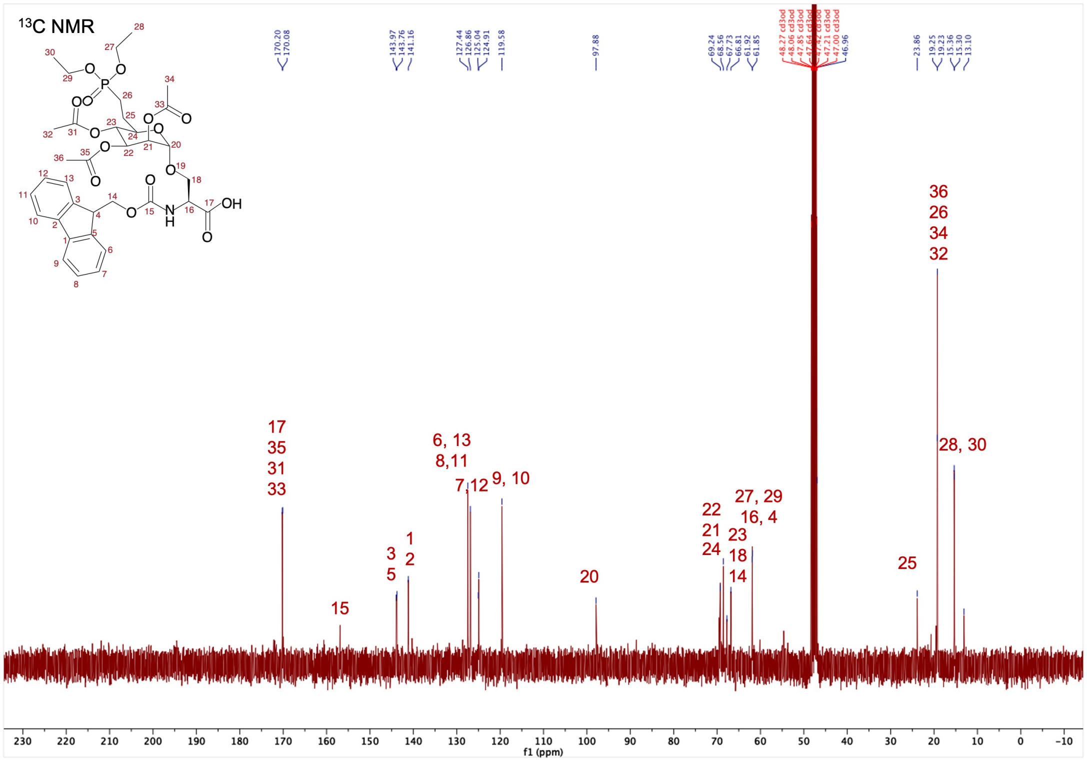

13C NMR
170.20 170.08
143.97 143.76 141.16
127.44 126.86 125.04 124.91 119.58
97.88
69.24 68.56 67.73 66.81 61.92 61.85
48.27 cd3od 48.06 cd3od 47.85 cd3od 47.21 cd3od 47.00 cd3od 46.96
23.86
19.25 19.23 15.36 15.30 13.10
17
35
31
33
15
3
5
1
2
6, 13
8,11
7, 12
9, 10
20
22
21
24
23
18
14
27, 29
16, 4
25
36
26
34
32
28, 30
230 220 210 200 190 180 170 160 150 140 130 120 110 100 90 80 70 60 50 40 30 20 10 0 −10
f1 (ppm)

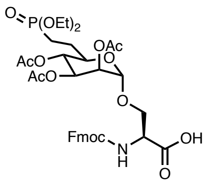
O=P(OEt)2
AcO
AcO
OAc
O
O
Fmoc
N
H
OH
O

$^{31}P$ NMR

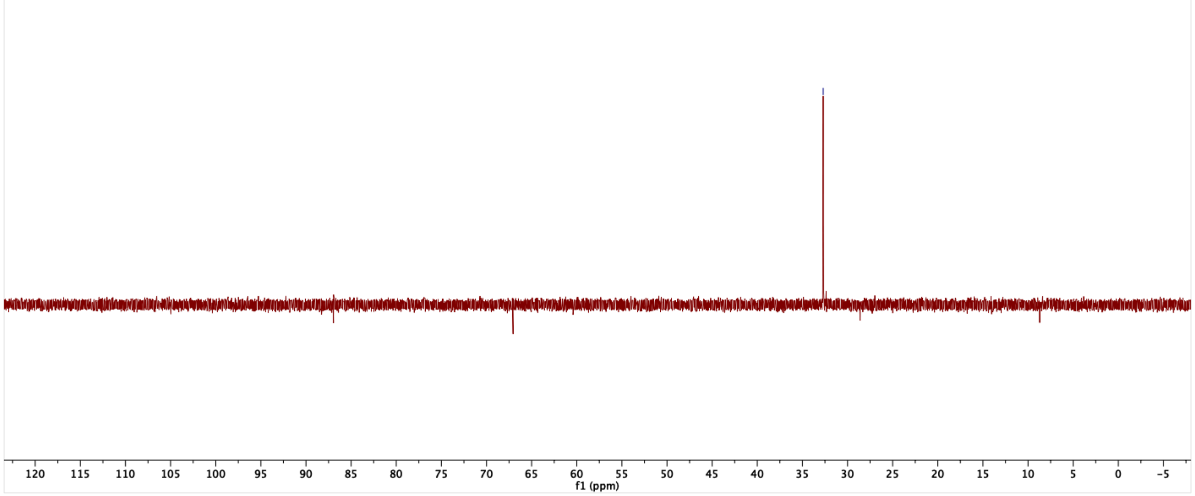
32.70
120 115 110 105 100 95 90 85 80 75 70 65 60 55 50 45 40 35 30 25 20 15 10 5 0 -5
f1 (ppm)
